# scDRMAE: integrating masked autoencoder with residual attention networks to leverage omics feature dependencies for accurate cell clustering

**DOI:** 10.1093/bioinformatics/btae599

**Published:** 2024-10-15

**Authors:** Tianjiao Zhang, Hongfei Zhang, Jixiang Ren, Zhenao Wu, Zhongqian Zhao, Guohua Wang

**Affiliations:** Department of Computer Science and Technology, College of Computer and Control Engineering, Northeast Forestry University, Harbin 150040, China; Department of Computer Science and Technology, College of Computer and Control Engineering, Northeast Forestry University, Harbin 150040, China; Department of Computer Science and Technology, College of Computer and Control Engineering, Northeast Forestry University, Harbin 150040, China; Department of Computer Science and Technology, College of Computer and Control Engineering, Northeast Forestry University, Harbin 150040, China; Department of Computer Science and Technology, College of Computer and Control Engineering, Northeast Forestry University, Harbin 150040, China; Department of Computer Science and Technology, College of Computer and Control Engineering, Northeast Forestry University, Harbin 150040, China; Department of Computer Science and Technology, Faculty of Computing, Harbin Institute of Technology, Harbin 150001, China

## Abstract

**Motivation:**

Cell clustering is foundational for analyzing the heterogeneity of biological tissues using single-cell sequencing data. With the maturation of single-cell multi-omics sequencing technologies, we can integrate multiple omics data to perform cell clustering, thereby overcoming the limitations of insufficient information from single omics data. Existing methods for cell clustering often only consider the differences in data patterns during the analysis of multi-omics data, but the dependencies between omics features of different cell types also significantly influence cell clustering. Moreover, the high dropout rates in scRNA-seq and scATAC-seq data can impact the performance of cell clustering.

**Results:**

We propose a cell clustering model based on a masked autoencoder, scDRMAE. Utilizing a masking mechanism, scDRMAE effectively learns the relationships between different features and imputes false zeros caused by dropout events. To differentiate the importance of various omics data in cell clustering, we dynamically adjust the weights of different omics data through an attention mechanism. Finally, we use the K-means algorithm for cluster analysis of the fused multi-omics data. On commonly used sets of 15 multi-omics datasets, our method demonstrates superior cell clustering performance on multiple metrics compared to other computational methods. In addition, when datasets exhibit varying degrees of dropout noise, our method shows better performance and stronger stability on multiple metrics compared to other methods. Moreover, by analyzing the cell clusters classified by scDRMAE, we identified several biologically significant biomarkers that have been validated, further confirming the effectiveness of scDRMAE in cell clustering from a biological perspective.

## 1 Introduction

Advances in single-cell RNA sequencing (scRNA-seq) technology have enabled biologists to measure whole-genome expression profiles at the single-cell level, effectively dissecting heterogeneous cell populations within complex samples that are inaccessible through traditional bulk sequencing methods. The rapid development of single-cell technologies provides a unique perspective for understanding cellular heterogeneity and dynamic changes within complex biological systems (Stuart and Satija 2019). Recently, the emergence of multimodal sequencing technologies such as CITE-seq (Stoeckius *et al.* 2017) and REAP-seq (Peterson *et al.* 2017), which combine transcriptomic sequencing with single-cell surface protein profiling, has allowed researchers to simultaneously study gene transcription levels and protein expression at the single-cell level. Specifically, CITE-seq uses existing single-cell sequencing technologies to quantify the abundance of cell surface proteins by counting antibody-derived tags (ADTs); REAP-seq integrates DNA-barcoded antibodies with scRNA-seq methods to measure both gene and cell surface protein expression levels. These methods offer high sensitivity for detecting low-abundance protein expressions, providing new possibilities for high-throughput single-cell analyses. In addition, the introduction of single-cell chromatin accessibility sequencing (scATAC-seq) (Buenostro *et al.* 2015) allows researchers to explore cell chromatin accessibility, revealing cell-type-specific gene expression regulatory mechanisms. Techniques such as SNARE-seq (Chen *et al.* 2019), which combine chromatin accessibility with gene expression analysis, offer new perspectives for analyzing RNA expression and chromatin accessibility simultaneously at the single-cell level. Recently, TEA-seq (Swanson *et al.* 2021) was introduced, integrating transcriptomic sequencing, single-cell surface protein profiling, and chromatin accessibility sequencing technologies, enabling simultaneous analysis of three types of omics data. These technologies provide biologists with a more comprehensive single-cell perspective on cellular system characterization, creating new opportunities for integrated cell analysis (Liu *et al.* 2023).

Cluster analysis is a crucial foundational step in most single-cell studies, allowing for the unsupervised identification of cell subpopulations, which provides key support for downstream differential expression analysis and complex disease research (Hu *et al.* 2024). Over the years, methods for analyzing single-cell RNA sequencing (scRNA-seq) data have continually evolved and expanded. Among these, the Tscan (Tschannen *et al.* 2018) method uses principal component analysis (PCA) and Gaussian mixture models (GMM) to cluster in a reduced dimensional space; Seurat (Satija *et al.* 2015) builds a k-nearest neighbors (KNN) graph based on Euclidean distances in PCA space, utilizing the Louvain/Leiden algorithm to achieve modular clustering of single cells. In contrast, SC3 (Kiselev *et al.* 2017) uses spectral clustering and aggregates individual clustering results calculated from different distance metrics, ultimately using hierarchical clustering to generate a comprehensive outcome. CASCC (Cai and Anastassiou 2024) aims to enhance clustering accuracy by utilizing gene co-expression features identified through an unsupervised adaptive attractor algorithm. The CTEC (Wang *et al.* 2024) approach combines two clustering results using a cross-tabulation to generate high-quality consensus clustering results. However, traditional single-cell clustering methods often perform poorly in feature extraction, typically only handling structurally simple datasets effectively. Recently, researchers have begun exploring the application of deep learning technologies in the clustering analysis of single-cell data. With continuous advancements in deep learning, various deep learning-based clustering methods have been developed to infer cell types from scRNA-seq data (Liu *et al.* 2024). Deep learning approaches like scDeepCluster (Tian *et al.* 2019) and DESC (Li *et al.* 2020) have achieved notable clustering results but often overlook the topological information between cells (Zhang *et al.* 2024a,b). To address this deficiency, Chen *et al.* proposed scGAC (Cheng and Ma 2022), which utilizes graph attention networks for cell clustering analysis. Simultaneously, Li *et al.* introduced scRISE (Xie *et al.* 2024), leveraging graph neural networks and iterative smoothing strategies to consider intercellular relationships for more accurate cell clustering. Moreover, scMAE (Fang *et al.* 2024) introduces a masking mechanism to perturb gene expression and uses a masked autoencoder to reconstruct the original data, thereby learning robust and informative cell representations. However, due to the limited information in single-omics data, especially in cases with numerous cell subtypes, clustering methods developed based on scRNA-seq often struggle to achieve ideal results. Therefore, further exploration of strategies and methods for integrating multi-omics data is expected to enhance the accuracy and effectiveness of single-cell cluster analysis.

In multi-omics data analysis, the biological information provided by different omics data is complementary and allows for features of cells from multiple perspectives (Zhang *et al.* 2024a,b). By integrating multi-omics data, it is possible to overcome the inherent limitations of single-omics data, thereby achieving more accurate cell clustering. In recent years, several clustering methods based on multi-omics data have been developed. For example, MoClust (Yuan *et al.* 2023) is a novel joint clustering framework that incorporates contrastive learning to enhance cluster compactness and separability, and accurately assesses the contribution of each omics type to the clustering objectives. scMNMF (Qiu *et al.* 2024) is an unsupervised method that jointly performs dimensionality reduction and clustering. It facilitates the discovery of cell types by allowing the dimensionality reduction feature selection and cell clustering to iteratively influence each other. scMLC (Chen *et al.* 2024) is a single-cell multi-modal Louvain clustering framework that partitions cell groups based on paired gene expression and chromatin accessibility data. Inspired by the principles of subspace clustering, Ren *et al.* proposed the scMCs (Ren *et al.* 2023) method, which minimizes redundancy between subspaces to achieve efficient parallel clustering of single-cell multi-omics data. In addition, scEMC (Hu *et al.* 2024) is a multi-modal clustering model for parallel scRNA-seq and scATAC-seq data. This model uses a denoising autoencoder based on ZINB loss, allowing the network to better fit the actual distribution of scRNA-seq data. However, we note that existing multi-omics clustering methods often do not fully consider the dependencies between internal features within different omics. In various cell types, dependencies between different omics features often exhibit significant variations, which could be informative for cell clustering. Moreover, scRNA-seq and scATAC-seq data are commonly associated with high noise and sparsity, along with dropout events (Kharchenko *et al.* 2014), leading to a prevalence of false zeros in the data. If the dependencies between different features can be effectively utilized, it would be possible to denoise the data, thereby obtaining more accurate biological information and improving the accuracy of cell clustering. In summary, integrating the feature dependency representations of different omics data promises to achieve more accurate cell clustering and data imputation.

Considering that previous methods have largely overlooked the dependencies between features and inspired by the Masked Autoencoders (MAE) (He *et al.* 2022), we propose the scDRMAE model, the first to apply MAE to the clustering analysis of single-cell multi-omics data. This model uses two parallel masked autoencoders to encode and reconstruct different omics data, thereby extracting the dependencies and omics information of various features. Given that the distribution characteristics of epigenomic data such as scATAC-seq are not yet clearly defined, we choose not to make assumptions about the distribution in the encoder’s low-dimensional space, maintaining the basic architecture of the MAE. To achieve better clustering results, we introduce a self-attention mechanism to dynamically allocate weights to concatenated different omics data. Furthermore, to prevent information loss during model training, we adopt a structure similar to ResNet (He *et al.* 2016), reintegrating the low-dimensional representation of scRNA-seq into the fused data processed by self-attention, thus avoiding the decline in cell clustering performance due to information loss. Finally, we introduced KL divergence to ensure effective interaction between the clustering module and the reconstruction module, to enhance clustering performance. To validate the effectiveness of the scDRMAE method, we conducted experiments on several real multi-omics datasets containing scRNA-seq data along with scATAC-seq or scADT-seq data, comparing scDRMAE with other advanced clustering methods. The results demonstrate that the scDRMAE model exhibits significant superiority in clustering performance.

## 2 Materials and methods

### 2.1 Datasets

We obtained 15 sets of real multi-omics datasets from GEO and previous research papers. These datasets can be categorized into two types based on the included omics: one type combines scRNA-seq data with scATAC-seq data, and the other combines scRNA-seq data with scADT-seq data. The first type includes 10 datasets, with data downloaded from the GEO database including the human cell line mixture (GSE126074) (Chen *et al.* 2019), mouse brain (GSE140203) (Moroney *et al.* 2020), and bone marrow mononuclear cells dataset BMMC (GSE194122) (Luecken *et al.* 2021). In addition, the processed Ma-2020 (Ma *et al.* 2020) dataset, which contains data from four batches, was obtained from a previous study by Gao *et al.* (Cao and Gao 2022). For the datasets combining scRNA-seq and scADT-seq, these include BMNC (available at https://github.com/satijalab/seurat-data), InHouse (GSE148665) (Wang *et al.* 2020), Stephenson (Stephenson *et al.* 2021), and PBMC10k (Jiang *et al.* 2023), with the Stephenson dataset divided into two sets, Ncl and Cambridge, based on the sequencing facility. The details of the dataset are shown in Table 1.

**Table 1.** The summary of datasets.^a^

Datasets	Cell count	Cell types	RNA_dim	Others_dim	Data types	Reference
human cell line mixture	1047	4	18 666	136 771	scRNA-seq, scATAC-seq	Chen *et al.* (2019)
Mouse brain	3293	19	21 127	428 041	scRNA-seq, scATAC-seq	Moroney *et al.* (2020)
MA-53	5692	22	21 478	340 341	scRNA-seq, scATAC-seq	Ma *et al.* (2020)
MA-54	10 709	22	21 478	340 341	scRNA-seq, scATAC-seq	Ma *et al.* (2020)
MA-55	9903	22	21 478	340 341	scRNA-seq, scATAC-seq	Ma *et al.* (2020)
MA-56	5927	22	21 478	340 341	scRNA-seq, scATAC-seq	Ma *et al.* (2020)
BMMC-24	6111	17	13 431	116 490	scRNA-seq, scATAC-seq	Luecken *et al.* (2021)
BMMC-49	4325	18	13 431	116 490	scRNA-seq, scATAC-seq	Luecken *et al.* (2021)
BMMC-41	8023	19	13 431	116 490	scRNA-seq, scATAC-seq	Luecken *et al.* (2021)
BMMC-36	1679	18	13 431	116 490	scRNA-seq, scATAC-seq	Luecken *et al.* (2021)
InHouse	1182	7	33 538	10	scRNA-seq, scADT-seq	Wang *et al.* (2020)
BMNC	30 672	27	17 009	25	scRNA-seq, scADT-seq	Lin *et al.* (2022)
Ncl	66 726	48	24 737	192	scRNA-seq, scADT-seq	Stephenson *et al.* (2021)
Cambridge	30 313	45	24 737	192	scRNA-seq, scADT-seq	Stephenson *et al.* (2021)
PBMC 10K	6661	6	33 538	14	scRNA-seq, scADT-seq	Jiang *et al.* (2023)

a RNA_dim represents the dimensionality information of the scRNA-seq data, while Others_dim indicates the dimensionality information of other omics data.

For the scRNA-seq data, we filtered out genes expressed in <1% of cells, selected 3000 highly variable genes, and performed log normalization and scaling; for the scATAC-seq data, we removed peaks present in <1% of cells, selected 3000 highly variable peaks, and conducted binarization, followed by feature transformation using TF-IDF (Martineau and Finin 2009); the scADT-seq data were also log normalized and standardized.

### 2.2 Evaluation metrics

This article evaluates the clustering performance of the model using two widely used metrics: the Adjusted Rand Index (ARI) and the Adjusted Mutual Information (AMI), as well as the Normalized Mutual Information (NMI).

ARI is a metric used to evaluate the similarity of clustering results. It assesses the quality of clustering by comparing the true labels of data points with the outcomes produced by the clustering algorithm, calculating their similarity. The value of ARI ranges from [−1, 1], where 1 indicates a perfect match, 0 represents the expected value of random clustering results, and −1 indicates a complete mismatch between the clustering results and the true labels. It is expressed as follows:

$$\mathrm{ARI}=\frac{2\left(ad-bc\right)}{\left(a+b\right)\left(b+d\right)+\left(a+c\right)\left(c+d\right)}.$$

In this formula, *a* represents the number of point pairs that belong to the same cluster in both the true and experimental scenarios, *b* represents the number of point pairs that belong to the same cluster in the true scenario but not in the experimental scenario, *c* represents the number of point pairs that do not belong to the same cluster in the true scenario but do belong to the same cluster in the experimental scenario, and *d* represents the number of point pairs that do not belong to the same cluster in either scenario.

NMI is a metric used to evaluate the similarity of clustering results, similar to ARI. It assesses the quality of clustering by comparing the information-theoretic measures between the clustering results and the true labels. The calculation of NMI is based on Mutual Information (MI), which considers the information shared between the true labels and the clustering results. After normalizing MI, the NMI is obtained, with a value range of [0, 1], where 1 indicates perfect matching and 0 indicates the expected value of random clustering results. It is expressed as follows:

$$\mathrm{NMI}=\frac{2MI\left(U;V\right)}{H\left(U\right)+H\left(V\right)}.$$

AMI is a metric used to measure the similarity of clustering results. AMI is an adjusted version of MI that accounts for the dependency between two random variables. The value of AMI ranges from [−1, 1], and it considers the degree of matching between clustering results and true labels while adjusting MI to address issues of imbalance and randomness. It is expressed as follows:

$$\mathrm{AMI}\left(U,V\right)=\frac{I\left(U;V\right)-E\left[I\left(U;V\right)\right]}{H\left(U\right)+H\left(V\right)-2E\left[I\left(U;V\right)\right]}.$$

### 2.3 Generate mask data

To enable the model to better learn the dependencies between features, we introduce randomness in feature expression across different omics data by randomly shuffling a subset of features within the original data matrix. Taking the scRNA-seq data $X_{\mathrm{RNA}-\mathrm{seq}}$ as an example, after shuffling within the features, we obtain the perturbed variant $X_{\mathrm{RNA}-\mathrm{seq}}^{s}$ of the original scRNA-seq data $\;X_{\mathrm{RNA}-\mathrm{seq}}$. According to existing research, the probability $P=\left(p_{1},p_{2},p_{3,\ldots ,}p_{n}\right)$ of dropout events occurring for each gene typically follows a Bernoulli distribution. To incorporate dropout events into the input data while enhancing the model’s ability to learn the dependencies between features, we generate a mask matrix $M_{\mathrm{RNA}-\mathrm{seq}}$ where each entry follows a Bernoulli distribution, expressed as:

$$M_{\mathrm{RNA}-\mathrm{seq}}∼\mathrm{Bernoulli}\left(P\right).$$

where *P* represents the probability of dropout events for different genes in different cells. Consequently, by combining the original scRNA-seq data $X_{\mathrm{RNA}-\mathrm{seq}}$, the perturbed data $X_{\mathrm{RNA}-\mathrm{seq}}^{s}$, and the mask matrix $M_{\mathrm{RNA}-\mathrm{seq}}$, we obtain the final masked data matrix $x_{\mathrm{RNA}-\mathrm{seq}}$ (Wan *et al.* 2022), expressed as:

$$x_{\mathrm{RNA}-\mathrm{seq}}{=X}_{\mathrm{RNA}-\mathrm{seq}}⊙\left(1-M_{\mathrm{RNA}-\mathrm{seq}}\right)+X_{\mathrm{RNA}-\mathrm{seq}}^{s}⊙M_{\mathrm{RNA}-\mathrm{seq}}.$$

Similarly, for other omics data, the masked data $x_{\mathrm{others}}$ is expressed as:

$$x_{\mathrm{others}}{=X}_{\mathrm{others}}⊙\left(1-M_{\mathrm{others}}\right)+X_{\mathrm{others}}^{s}⊙M_{\mathrm{others}}.$$

### 2.4 Mask autoencoder

**Figure 1.**
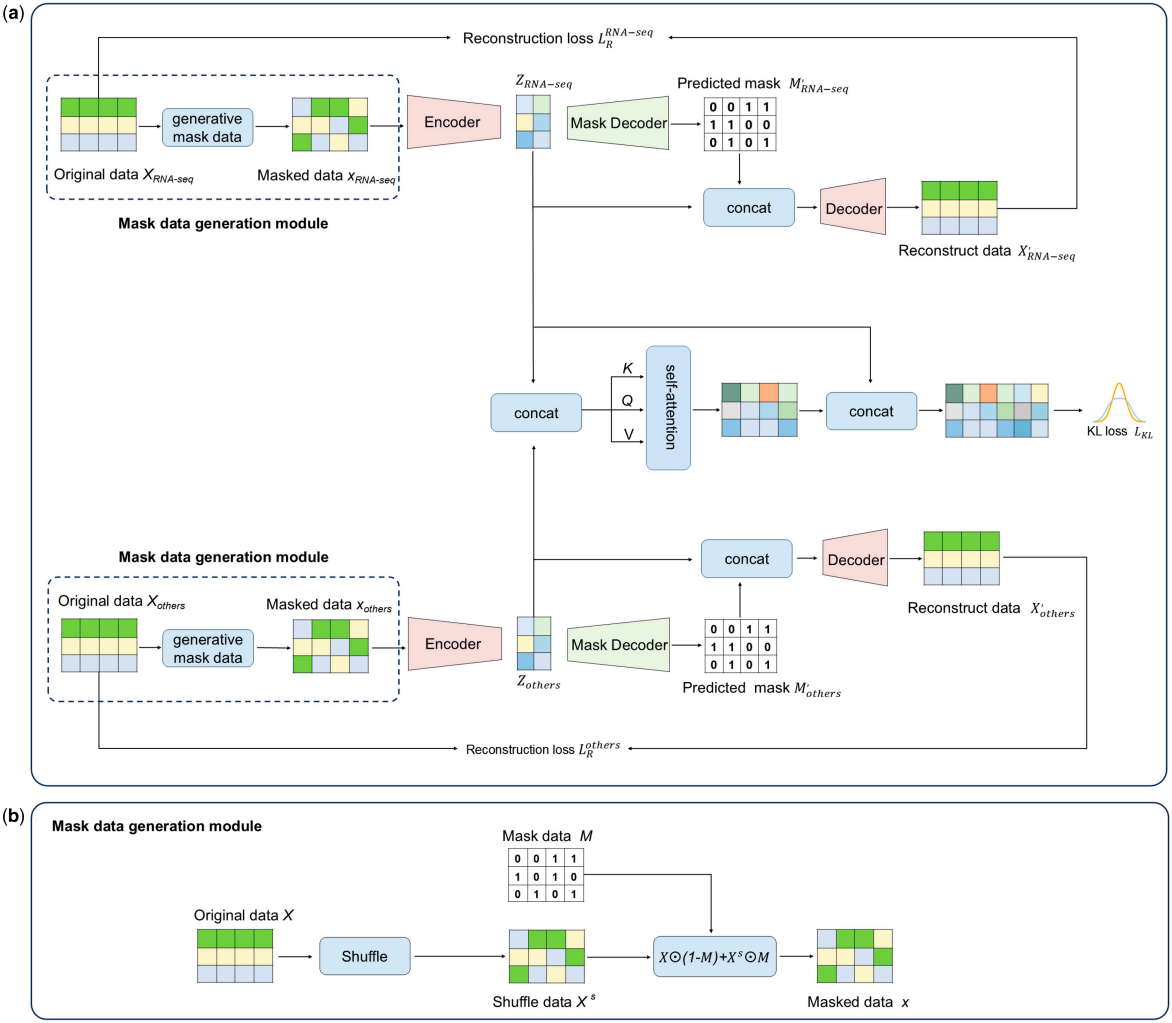
scDRMAE Framework. (a) Overall framework of scDRMAE. (b) Masked data generation module.

The architecture of the scDRMAE is illustrated in Fig. 1. It consists of two sets of parallel encoders, a mask decoder, and a decoder. The encoders are used to reduce the dimensionality of the masked data from different omics, projecting it into a low-dimensional space, represented as:

$$Z_{\mathrm{RNA}-\mathrm{seq}}=f_{e}^{\mathrm{RNA}-\mathrm{seq}}\left(x_{\mathrm{RNA}-\mathrm{seq}}\right);Z_{\mathrm{others}}=f_{e}^{\mathrm{others}}\left(x_{\mathrm{others}}\right).$$

where $f_{e}^{\mathrm{RNA}-\mathrm{seq}}\left(.\right)$ and $f_{e}^{\mathrm{others}}\left(.\right)$ denote the encoders for different omics, used to project $x_{\mathrm{RNA}-\mathrm{seq}}$ and $x_{\mathrm{others}}$ into a low-dimensional space. The mask decoder, $f_{m}^{\mathrm{RNA}-\mathrm{seq}}\left(.\right)\;$ and $f_{m}^{\mathrm{others}}\left(.\right)$, is responsible for predicting the corresponding mask matrices for different omics data from the low-dimensional representations$\;Z_{\mathrm{RNA}-\mathrm{seq}\;}$ and $Z_{\mathrm{others}}$, represented as:

$$M_{\mathrm{RNA}-\mathrm{seq}}^{\prime }=f_{m}^{\mathrm{RNA}-\mathrm{seq}}\left(Z_{\mathrm{RNA}-\mathrm{seq}}\right);M_{\mathrm{others}}^{\prime }=f_{m}^{\mathrm{others}}\left(Z_{\mathrm{others}}\right).$$

Here, $M_{\mathrm{RNA}-\mathrm{seq}}^{\prime }$ and $M_{\mathrm{others}}^{\prime }$ are the predicted mask matrices for different omics from the mask decoder. By using binary cross-entropy loss as the mask prediction loss $L_{M}$, the model can learn the dropout events in the omics data. This is expressed as follows:

$$\begin{array}{l} L_{M}^{\mathrm{RNA}-\mathrm{seq}}=-\frac{1}{n*g}\sum_{i=1}^{n}\sum_{j=1}^{g}[M_{\mathrm{RNA}-\mathrm{seq}}^{ij}\log M_{\mathrm{RNA}-\mathrm{seq}}^{ij^{\prime }}+(1–M_{\mathrm{RNA}-\mathrm{seq}}^{ij})\log\left(1{–M}_{\mathrm{RNA}-\mathrm{seq}}^{ij^{\prime }}\right)];\\ L_{M}^{\mathrm{others}}=-\frac{1}{n*p}\sum_{i=1}^{n}\sum_{j=1}^{p}\left[M_{\mathrm{others}}^{ij}\log M_{\mathrm{others}}^{ij^{\prime }}+(1-M_{\mathrm{others}}^{ij})\log\left(1{-M}_{\mathrm{others}}^{ij^{\prime }}\right)\right]\\ L_{M}=L_{M}^{\mathrm{RNA}-\mathrm{seq}}+L_{M}^{\mathrm{others}} \end{array}.$$

where $L_{M}^{\mathrm{RNA}-\mathrm{seq}}$ and $L_{M}^{\mathrm{others}}$ represent the mask prediction losses for different omics, with *n* indicating the number of cells, and *g* and *p* representing the feature dimensions of different omics data. $M_{\mathrm{RNA}-\mathrm{seq}}^{ij}$ and $M_{\mathrm{others}}^{ij}$ denote the true mask matrices, while $M_{\mathrm{RNA}-\mathrm{seq}\;}^{ij^{\prime }}$ and$\;M_{\mathrm{others}}^{ij^{\prime }}$ represent the predicted mask matrices from the mask decoder.

We combine the predicted mask matrices for different omics with the low-dimensional representations and pass them to the decoder to reconstruct the different omics data, expressed as:

$$\begin{array}{c} X_{\mathrm{RNA}-\mathrm{seq}}^{\prime }=f_{d}^{\mathrm{RNA}-\mathrm{seq}}\left(\left[M_{\mathrm{RNA}-\mathrm{seq}}^{ij^{\prime }},Z_{\mathrm{RNA}-\mathrm{seq}}\right]\right);\\ X_{\mathrm{others}}^{\prime }=f_{d}^{\mathrm{others}}\left(\left[M_{\mathrm{others}}^{ij^{\prime }},Z_{\mathrm{others}}\right]\right) \end{array}$$

where $X_{\mathrm{RNA}-\mathrm{seq}}^{\prime }$ and $X_{\mathrm{others}}^{\prime }$ are the reconstructed data for different omics. We calculate the mean squared error between the reconstructed data and the original data as the reconstruction $L_{R}$, while assigning different weights to damaged and undamaged features, expressed as:

$$\begin{array}{c} L_{R}^{\mathrm{RNA}-\mathrm{seq}}=-\frac{1}{n*g}\sum_{i=1}^{n}\sum_{j=1}^{g}{W_{ij}^{\mathrm{RNA}-\mathrm{seq}}\left|X_{\mathrm{RNA}-\mathrm{seq}}-X_{\mathrm{RNA}-\mathrm{seq}}^{\prime }\right|}^{2};\\ \begin{array}{c} L_{R}^{\mathrm{others}}=-\frac{1}{n*p}\sum_{i=1}^{n}\sum_{j=1}^{p}{W_{ij}^{\mathrm{others}}\left|X_{\mathrm{others}}-X_{\mathrm{others}}^{\prime }\right|}^{2};\\ L_{R}=L_{R}^{\mathrm{RNA}-\mathrm{seq}}+L_{R}^{\mathrm{others}} \end{array} \end{array}$$

where $L_{R}^{\mathrm{RNA}-\mathrm{seq}}\;\mathrm{and}\;L_{R}^{\mathrm{others}}$ represent the reconstruction losses for different omics data, and $W_{ij}^{\mathrm{RNA}-\mathrm{seq}}$ and $W_{ij}^{\mathrm{others}}$ denote the reconstruction weights for disturbed and undisturbed features in scRNA-seq data and other omics data, respectively.

### 2.5 Multi-omics data integration

To further fuse the low-dimensional representations of different omics, we first concatenate the low-dimensional representations of different omics, represented as:

$$Z=\left[Z_{\mathrm{RNA}-\mathrm{seq},}Z_{\mathrm{others}}\right].$$

Then, we apply an attention mechanism to transform *Z*, allowing it to focus more on the features that are important for clustering results. We will adopt a strategy similar to that used in transformers (Vaswani *et al.* 2017), mapping *Z* into three different feature subspaces:

$$K=ZW_{1};Q=ZW_{2};V=ZW_{3},$$

where $W_{1}$, $W_{2},\;$ and${\;W}_{3}$ are the weight matrices used for mapping transformations, and *K*, *Q*, and *V* represent the three feature encodings of *Z* after linear transformations. Next, we compute the global relationship matrix *W* using *K* and *Q*:

$$W=\mathrm{softmax}\left(\frac{QK^{T}}{\sqrt{d}}\right),$$

where *d* represents the feature dimension of *Z*. Subsequently, we enhance the features *V* using *W* to obtain the enhanced feature $\bar{Z}$:

$$\bar{Z}=WV+b,$$

where *b* is the bias term. Based on past experiences, scRNA-seq data often provides richer information during cell clustering processes. To prevent degradation issues during model training, which may lead to the loss of scRNA-seq information critical for clustering, we are inspired by ResNet and fuse the scRNA-seq feature $Z_{r}$ with $\bar{Z}$ again, to acquire a richer feature representation $\hat{Z}$, expressed as:

$$\hat{Z}=\left[Z_{\mathrm{RNA}-\mathrm{seq}},\bar{Z}\right].$$

To further optimize the fused feature $\hat{Z}$ and allow for information interaction between different omics’ MAE frameworks, we introduce the KL divergence loss as a constraint. This helps compact similar cells and separate different types of cells. Referencing previous research (Lin *et al.* 2022, Meitz *et al.* 2023), we describe the similarity between cell i  and cell j using a *t*-distribution:

$$q_{ij}=\frac{{\left(1+∥{\hat{Z}}_{i}-{\hat{Z}}_{j}{∥}^{2}\right)}^{-1}}{\sum_{l\neq i}\;{\left(1+∥{\hat{Z}}_{i}-{\hat{Z}}_{j}{∥}^{2}\right)}^{-1}},$$

where ${\hat{Z}}_{i}$ and ${\hat{Z}}_{j}$ represent the encoded representations of cell *i* and cell *j*, respectively, and $q_{ij}$ denotes the soft assignment representing the pairwise similarity between cell *i* and cell *j*. The target distribution $p_{ij}$ is constructed based on $q_{ij}$, normalized to enhance the affinity between cells with high similarity and reduce the affinity between cells with low similarity. The calculation process is as follows:

$$p_{ij}=\frac{q_{ij}^{2}/\sum_{i=1}^{n}\;q_{ij}}{\sum_{i\neq i}\;\left(q_{il}^{2}/\sum_{l\neq i}\;q_{il}\right)}.$$

After obtaining the two distributions, we impose a constraint on the features by calculating the KL divergence between the two distributions, thus improving clustering performance:

$$L_{kl}=KL(P||Q)=\sum_{i}\;\sum_{j}\;p_{ij}l\mathrm{og}\frac{p_{ij}}{q_{ij}}.$$

### 2.6 Clustering analysis

Finally, the fused multi-omics data is subjected to cluster analysis using the K-means method.

### 2.7 Parameter configuration

In the model implementation process, the structures of different omics encoders were sequentially connected to a Dropout layer, a 256D linear layer, a layer normalization layer, a Mish activation layer, a 64D linear layer, another layer normalization layer, and finally a 64D linear layer, progressively mapping the input gene features into more expressive feature representations. To enable the encoder to fully learn the distinct omics characteristics, we retained the asymmetric structure of the MAE model for the decoder, using only a fully connected layer as the decoder.

## 3 Results

Our experiments were conducted on a server operating on a Linux system, equipped with an NVIDIA A100 GPU and 80 GB of memory. During the experimental process, due to variations in dataset size and dimensionality, we configured different hyperparameters for each dataset to achieve optimal clustering results. Specifically, for the InHouse dataset, we set the epochs to 20 with a learning rate of 0.001; for the human cell line mixture dataset, the epochs were set to 50 with a learning rate of 0.001; for the PBMC10K dataset, we configured the epochs to 20 with a learning rate of 0.002; and for other datasets, the epochs were set to 100 with a learning rate of 0.002 in pursuit of optimal solutions. All comparative methods were processed according to the data processing workflows specified in the original articles, adhering to the descriptions provided in their respective official repositories, with parameter settings aligned with the default configurations.

### 3.1 The comparison of clustering performance of scDRMAE with existing methods

**Figure 2.**
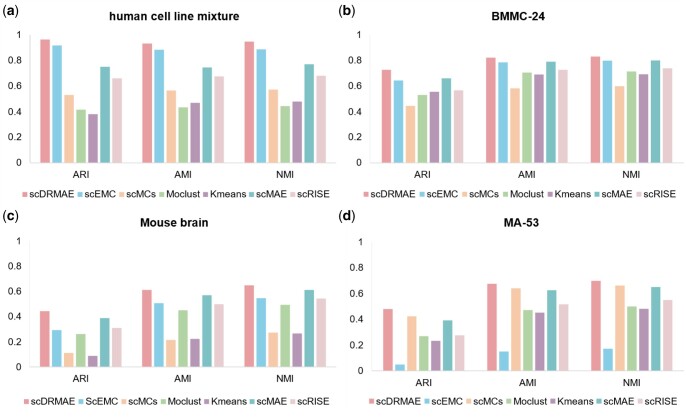
Comparison of clustering performance of different methods on four scRNA-seq and scATAC-seq datasets. (a) Comparison of clustering metrics on the human cell line mixture dataset, (b) comparison of clustering metrics on the BMMC-24 dataset, (c) comparison of clustering metrics on the Ma-53 dataset, and (d) comparison of clustering metrics on the mouse brain dataset.

We first compared the scDRMAE method with six other algorithms on ten multi-omics datasets that include scRNA-seq and scATAC-seq data. Figure 2 displays the clustering performance metrics for these methods on four datasets, including AMI, NMI, and ARI. For clustering results on all datasets, please refer to Supplementary Tables S1–S6. The results indicate that scDRMAE outperforms other comparative methods for both NMI and AMI metrics. For the ARI metric, scDRMAE achieves optimal results on all datasets except for the MA-56 dataset, where it slightly lags behind scMCs. Furthermore, we found that the performance of most multi-omics clustering methods surpasses that of single-omics clustering methods. This outcome further validates the effectiveness of using multi-omics data for cell clustering, attributable to the richer cellular heterogeneity information provided by multi-omics data compared to single-omics data, which helps enhance the performance of cell clustering. Therefore, integrating multi-omics data for analysis is essential.

**Figure 3.**
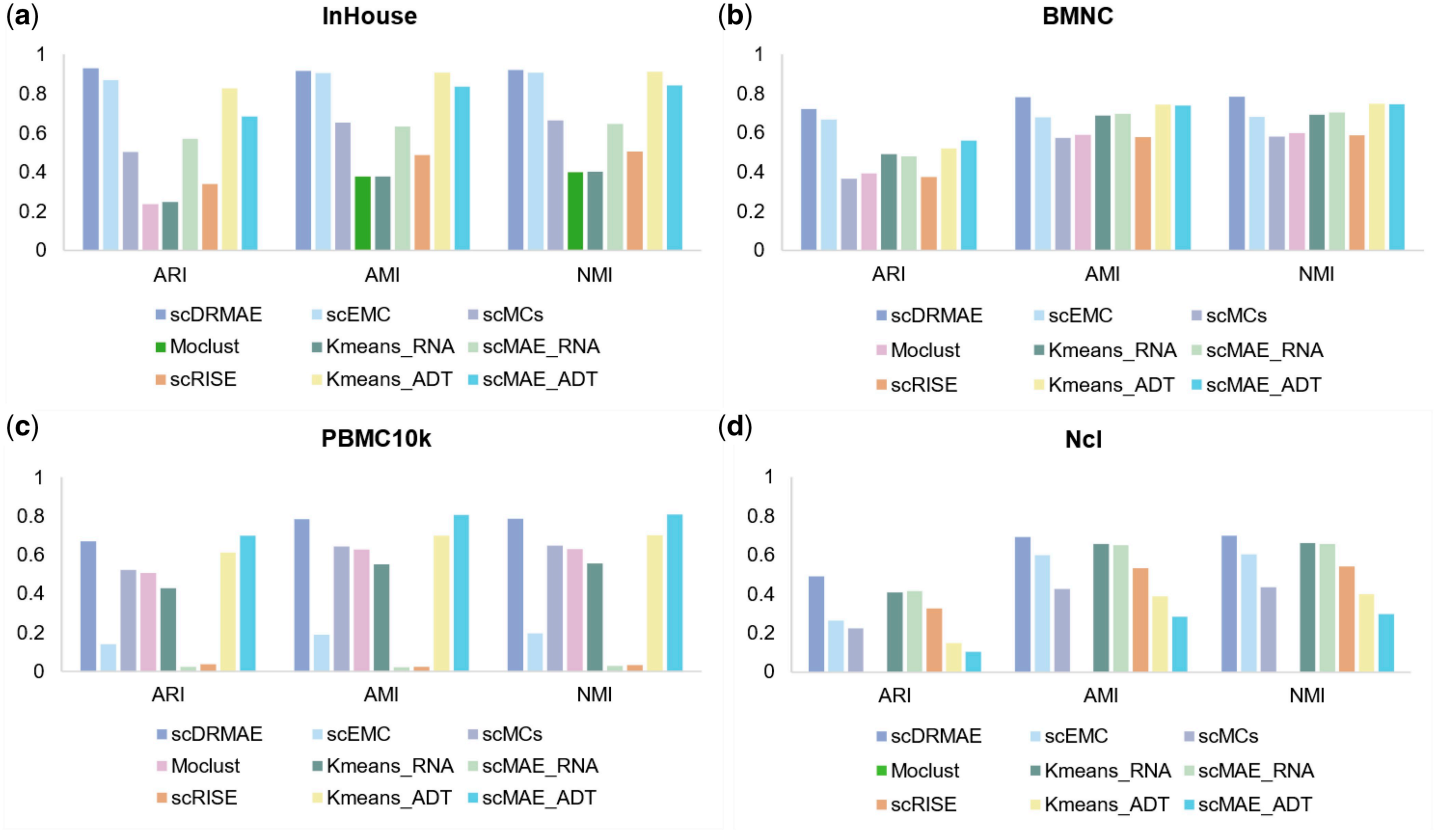
Comparison of clustering performance of different methods on four scRNA-seq and scADT-seq datasets. (a) Comparison of clustering metrics on the InHouse dataset, (b) comparison of clustering metrics on the BMNC dataset, (c) comparison of clustering metrics on the PBMC10k dataset, and (d) comparison of clustering metrics on the Ncl dataset.

To assess the generalization capability of the scDRMAE model, we conducted experiments on five additional multi-omics datasets composed of scRNA-seq and scADT-seq data. Figure 3 displays the clustering performance of different methods on this type of dataset. Apart from the PBMC10k dataset, where scDRMAE achieved second place, it exhibited the best performance on the remaining four datasets. In addition, Ncl and Cambridge are two multi-batch datasets, each covering peripheral blood mononuclear cell samples from 12 individuals, containing 48 and 45 cell subtypes, respectively. Figure 3c and Supplementary Tables S4–S6 show the clustering results of different methods on these two datasets, demonstrating the advantages of the scDRMAE method in handling multi-batch datasets.

Interestingly, during the execution of various single-omics clustering methods, we observed that in datasets with a larger number of cell types (over 40), the performance of clustering using single scADT-seq data was significantly inferior to that using single scRNA-seq data. Conversely, in datasets with fewer cell subtypes, the clustering effectiveness using scADT-seq data was notably superior to that using scRNA-seq data. This result indicates that surface proteins can effectively differentiate broad categories of cells, but due to their lower feature dimensionality and limited information content, they perform poorly in distinguishing cell subtypes. Meanwhile, scRNA-seq data provide a more comprehensive characterization of cell states, thus demonstrating greater advantages in clustering cell subtypes.

**Figure 4.**
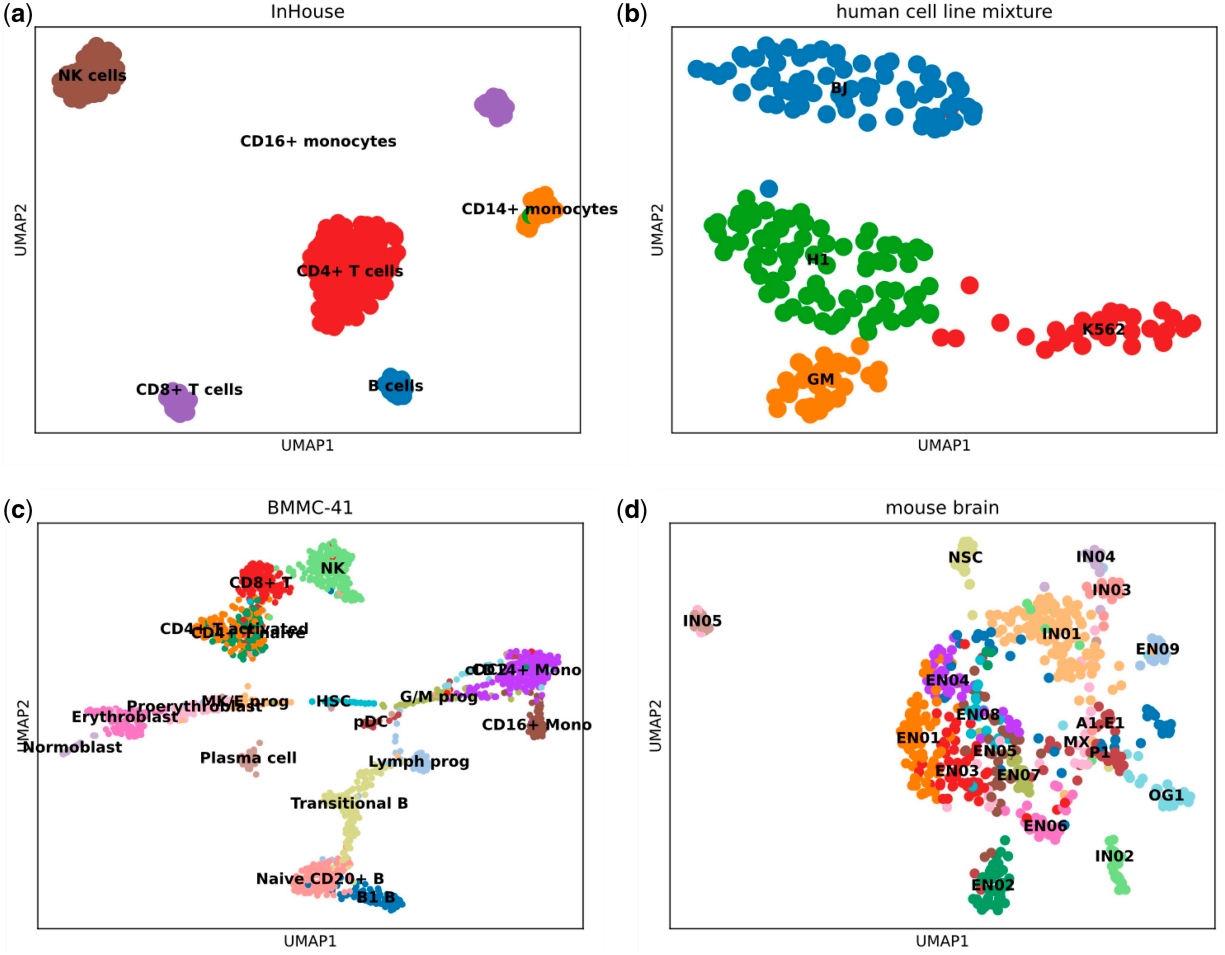
UMAP visualization of four datasets after dimensionality reduction using scDRMAE. (a) UMAP plot of the InHouse dataset, (b) UMAP plot of the human cell line mixture dataset, (c) UMAP plot of the BMMC-41 dataset, and (d) UMAP plot of the mouse brain dataset.

To visually demonstrate the clustering performance of scDRMAE, we visualized the low-dimensional representations of different datasets, as shown in Fig. 4. Figure 4a displays the UMAP plot for the InHouse dataset, which consists of six cell types; Fig. 4b shows the UMAP plot for the human cell line mixture dataset, which is composed of four cell types. Meanwhile, Fig. 4c and d present the UMAP plots for the mouse brain and MA-53 datasets, which contain 19 and 22 cell types, respectively. Overall, the separation of different major cell categories is quite distinct across the four datasets, particularly in the human cell line mixture and InHouse datasets, which have fewer cell subtypes. The relatively poorer separation in the mouse brain and MA-53 datasets compared to the first two is anticipated, as these datasets include a larger number of cell subtypes, and the expression patterns of features between different subtypes are often very similar, leading to blurred boundaries between different cell subgroups and less distinct separation. Overall, the low-dimensional representations produced by scDRMAE effectively differentiate various types of cell populations, demonstrating the superior clustering performance of scDRMAE.

### 3.2 The clustering performance of scDRMAE under high dropout noise datasets

**Figure 5.**
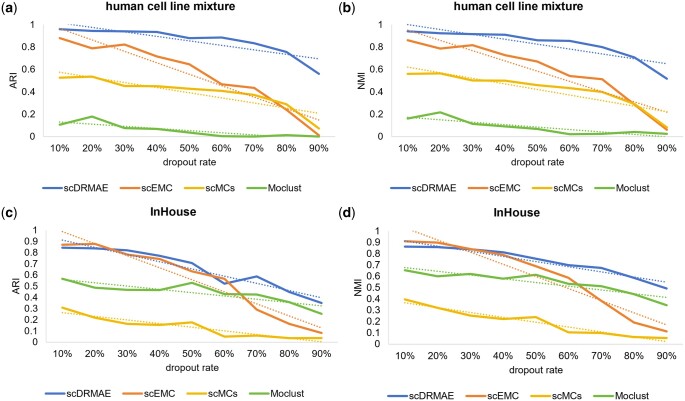
Clustering metrics of various methods at different dropout rates across four datasets. (a) ARI of different methods on the human cell line mixture dataset, (b) NMI of different methods on the human cell line mixture dataset, (c) ARI of different methods on the InHouse dataset, and (d) NMI of different methods on the InHouse dataset.

To demonstrate the noise resistance and the ability of scDRMAE to learn dependencies between features, we simulated dropout events at different probabilities by combining different test sets with 0–1 masking matrices at varying probabilities. Figure 5 and Supplementary Figure S1 illustrate the clustering performance of various methods under different dropout rates.

**Figure 6.**
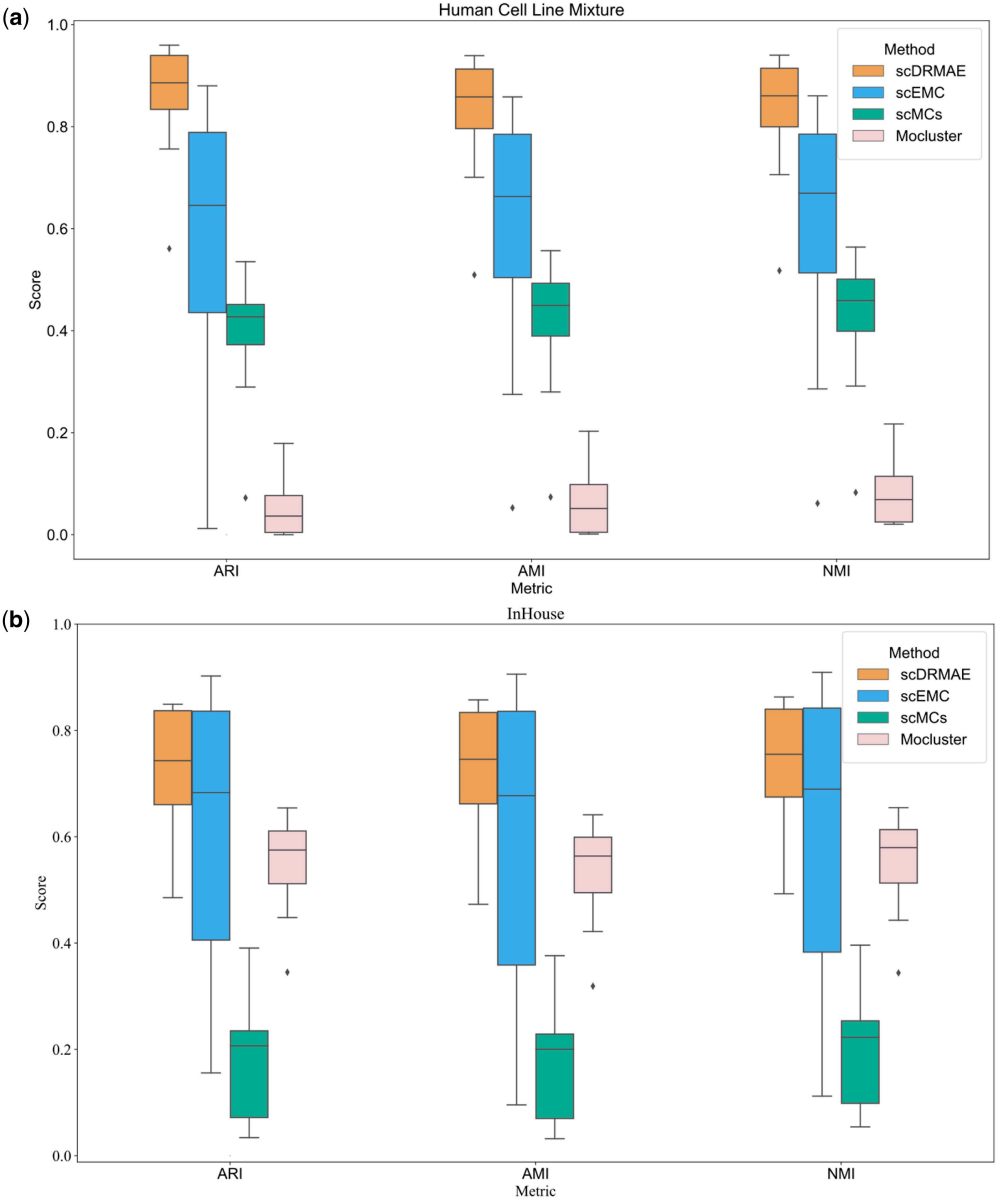
Box plots of clustering metrics for different methods on two datasets. (a) Box plot for the human cell line mixture dataset. (b) Box plot for the InHouse dataset.

Comparing the clustering metrics of different methods across various dropout probabilities, it can be observed that as the dropout probability increases, the clustering performance of all methods declines, with scEMC exhibiting the largest fluctuations. Notably, on the cell_line dataset, when the dropout rate rises to 90%, scDRMAE still achieves an ARI of 0.56, while the ARI, AMI, and NMI of other methods like scEMC are nearly zero. Figure 6 presents box plots of various clustering metrics for different methods. Observing Fig. 6a and b, it can be seen that scDRMAE, scMCs, and Moclust exhibit smaller fluctuations, but across different dropout rates, scDRMAE consistently outperforms these two methods. The superior performance of scDRMAE is attributed to its unique masking prediction mechanism, which enables it to learn the dependencies between different features and effectively identify the occurrence of dropout events. Therefore, even under extremely high dropout rates, scDRMAE still maintains good performance.

### 3.3 scDRMAE improves cell clustering performance by imputing scRNA-seq data

**Figure 7.**
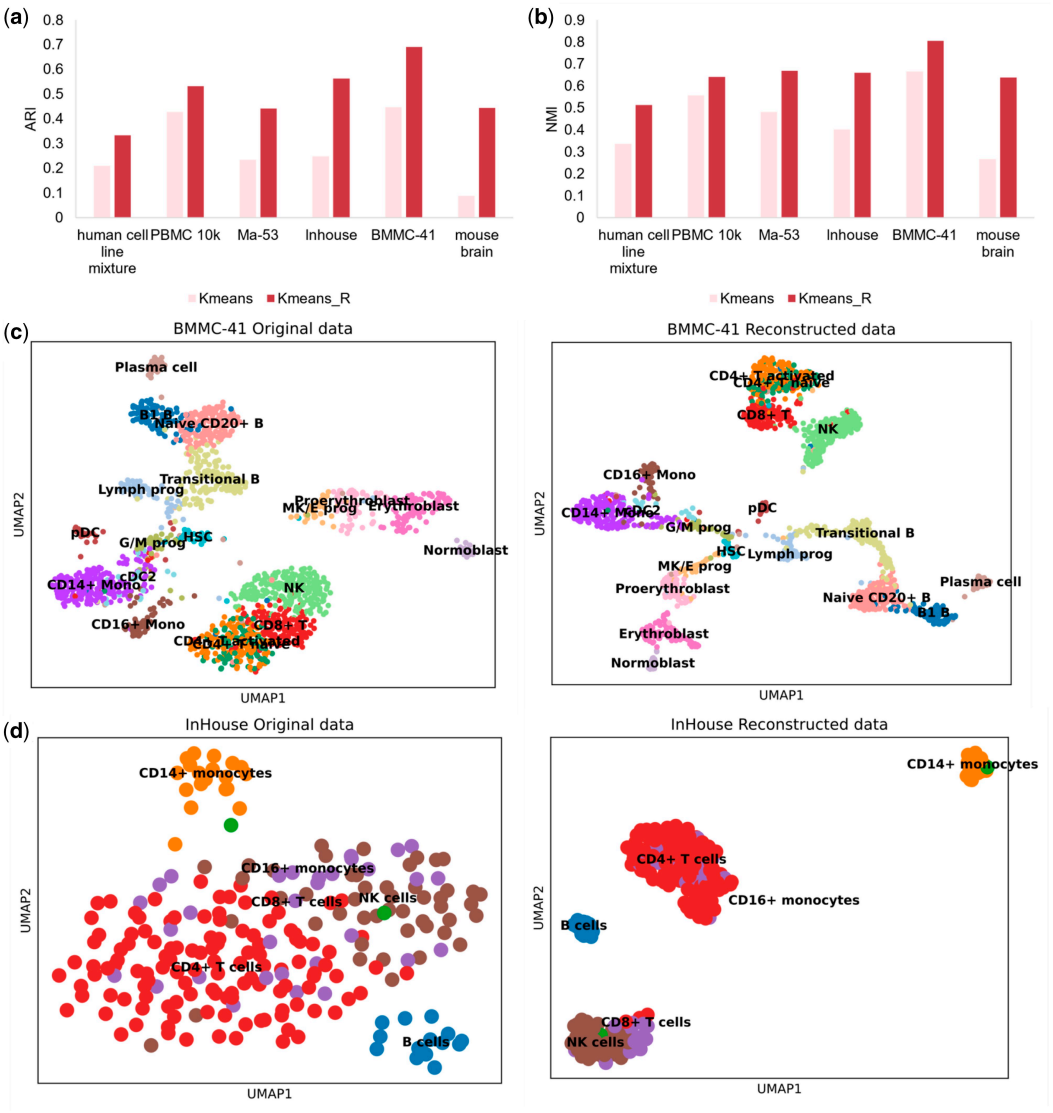
Performance of scDRMAE for data imputation. (a) ARI before and after imputation on different datasets. (b) AMI before and after imputation on different datasets. (c) UMAP plots of the BMMC-41 dataset showing scRNA-seq data before and after imputation, annotated with true labels, where the left plot is the original data and the right plot is the imputed data. (d) UMAP plots of the mouse brain dataset showing scRNA-seq data before and after imputation using scDRMAE, annotated with true labels, where the left plot is the original data and the right plot is the imputed data.

To further validate the capability of scDRMAE in learning feature dependencies and the impact of data imputation on clustering, we conducted clustering analysis on data before and after imputation. Figure 7 shows a comparison of the K-means clustering performance on data imputed by scDRMAE versus the performance on nonimputed data. Observing Fig. 7a, b and Supplementary Fig. S2a, it is evident that the clustering metrics on various datasets are improved after imputation compared to before imputation.

To more vividly demonstrate the impact of scDRMAE’s imputation mechanism on clustering, we plotted the UMAP maps of the original and reconstructed data, as shown in Fig. 7c, d, Supplementary Fig. S2b and c. The images on the left display the distribution of the original data, where many cells are mixed together and the boundaries between different cell types are unclear. In contrast, the reconstructed data effectively separates different types of cells, clustering cells of the same type together. This phenomenon highlights the advantages brought by imputation, making the distribution differences between different cell types more pronounced, thereby enhancing the effectiveness of cell clustering. In summary, the scDRMAE method demonstrates strong performance in data imputation, and using the imputed multi-omics data can effectively improve clustering results.

### 3.4 Identifying biomarkers in different cell types

Marker genes can be used to annotate specific cell types; by studying their expression patterns, we can elucidate cellular heterogeneity and potential gene regulatory mechanisms. To further demonstrate the accuracy of the scDRMAE method in cell clustering, we used the nonparametric Wilcoxon rank-sum test to identify the top three differential features (genes, surface proteins, and open chromatin regions) for each predicted cluster and validated their cell-type specificity using databases such as GeneCards (Safran *et al.* 2010) and GenBank (Benson *et al.* 2013). Supplementary Figure S3a displays the top three differential genes in cell clusters classified by scDRMAE within the InHouse dataset. We checked these differential genes in GeneCards and found that the genes selected from different clusters have biological significance and are strongly associated with certain types of cells, further confirming the accuracy of cell clustering by scDRMAE.

For example, in cluster 0, CD8B has been validated as a marker gene for CD8+ T cells (Guo *et al.* 2018), while IL7R is a marker for Naïve and memory T cells (Sinha *et al.* 2018). In cluster 1, the GNLY gene is confirmed as a marker gene for natural killer (NK) cells (Zhang *et al.* 2018). The NKG7 gene, crucial for the cytotoxic degranulation of NK and CD8 T cells and the activation and pro-inflammatory responses of CD4 T cells (Turman *et al.* 1993), is also proven to be a marker for NK cells. In addition, the GZMB gene, which encodes granzyme B belonging to the serine protease S1 family (Zhou *et al.* 2020), is secreted by NK cells and cytotoxic T lymphocytes (CTLs) as a precursor and processed into an active protease through proteolytic cleavage, inducing apoptosis in target cells. In cluster 2, S100A8 and S100A9, belonging to the S100 family of calcium-binding proteins, are primarily expressed in neutrophils and monocytes and play key roles in regulating various inflammatory responses and related diseases, thus serving as markers for monocytes. In cluster 3, the predicted marker genes CD79A and CD79B have been established as markers for B cells (Huse *et al.* 2022). The CD74 protein, produced by the CD74 gene, is a type II transmembrane glycoprotein involved in regulating the survival signaling pathways of B cells (David *et al.* 2022). In addition, the B lymphocyte antigen CD20, encoded by the MS4A1 gene, is expressed at nearly all stages of B cell development, making it one of the most commonly used TIL-B biomarkers to date (Liu *et al.* 2020). In cluster 4, the TRAC gene has been previously identified as being enriched in γδ T cells. Lastly, in cluster 5, CCL5 has been recognized as playing a significant role in CD8+ T cells (Appay and Rowland-Jones 2001), while NKG7 can also identify CD8+ T cells to a certain extent (Malarkannan 2020).

We also conducted rank-based analysis on the proteomics data from the InHouse dataset. Supplementary Figure S3b shows the top three marker proteins for the different clusters identified in the InHouse dataset. In cluster 0, CD8a was also confirmed as a marker for CD8+ T cells (Wang *et al.* 2009). In cluster 1, CD56 was identified as a fundamental biomarker for natural killer (NK) cells (Picard *et al.* 2022). In addition, subtypes of NK cells can be further differentiated based on the relative expression levels of CD16 and CD56 (Poli *et al.* 2009). In cluster 2, CD14 was determined to be a marker for monocytes, capable of triggering intracellular signaling in response to bacterial contact (Wu *et al.* 2019). CD11c is also predominantly expressed in monocytes, further supporting the characteristics of this cell type. In cluster 3, CD19, predicted as a biomarker, is important for both normal and neoplastic B cells as well as follicular dendritic cells (Nadler *et al.* 1983). CD19 plays a crucial role in setting the threshold for internal B cell signal transduction by regulating both B cell receptor-dependent and -independent signaling pathways. In cluster 4, the discovery of CD4 protein highlights its importance as a marker for CD4+ T cells (Beura *et al.* 2019). CD4 protein is located on the surface of CD4+ T cells, while molecules such as CD3, CD127, and CD154 are also predominantly expressed in T cells, further validating the characteristics of T cells. In cluster 5, both CD8a and CD154 were proven to be biomarkers for T cells (Chattopadhyay *et al.* 2005). In addition to performing differential analysis on the InHouse dataset, we also conducted differential analysis on three other datasets: human cell line mixture, mouse brain, and BMMC-41, and visualized the differentially expressed genes across the different clusters, as shown in Supplementary Figures S4–S10.

**Figure 8.**
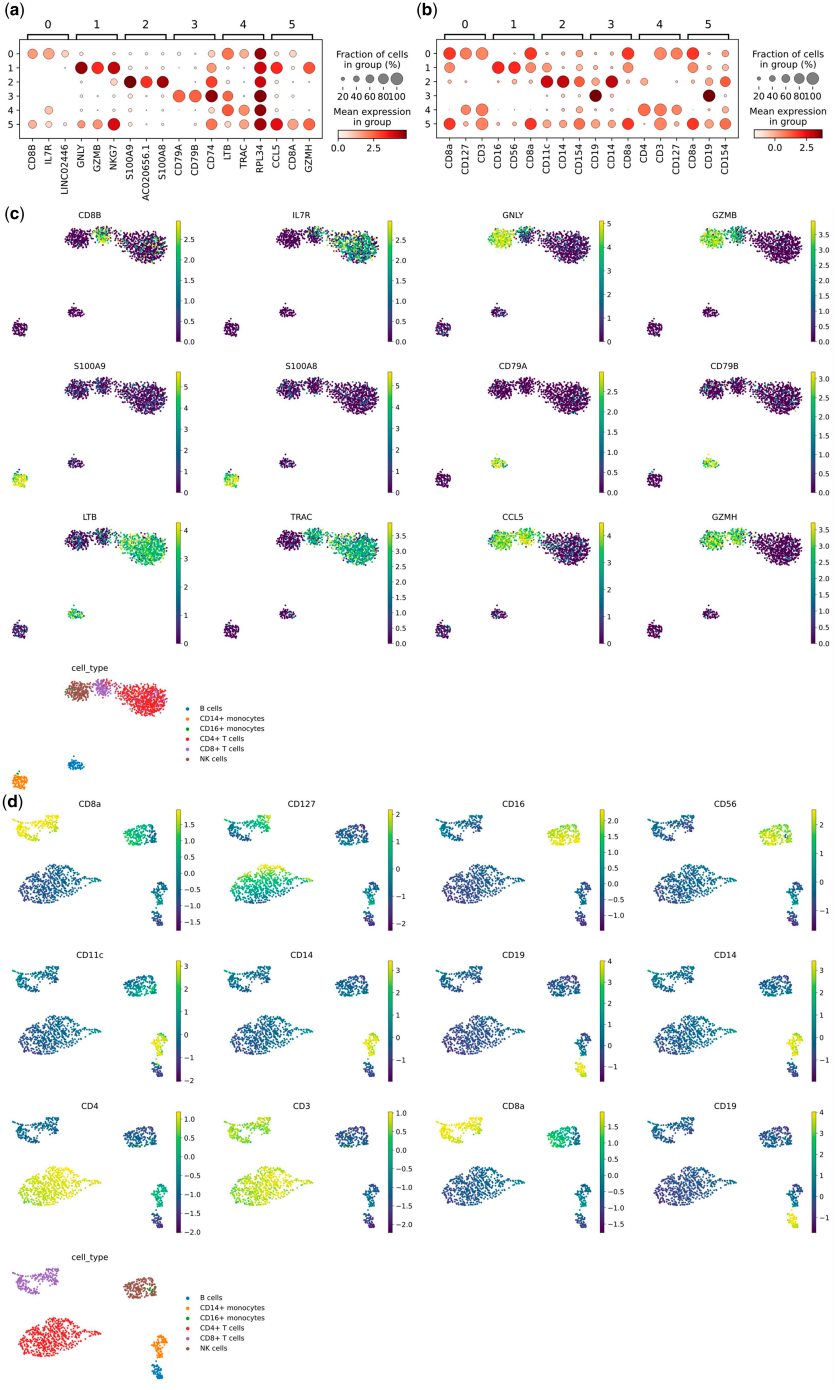
Differential genes and their visualization among different clusters. (a) Bubble plot showing the top three differential genes identified by scDRMAE across different clusters. (b) Bubble plot showing the top three differential proteins identified by scDRMAE across different clusters. (c) Visualization of differential genes among different clusters on a UMAP plot. (d) Visualization of differential proteins among different clusters on a UMAP plot.

During the differential analysis, we found that although the two selected differential genes S100A8 and S100A9 in cluster 2 can identify both monocytes and neutrophils, it is often difficult to distinguish these two cell types using just these markers. However, when we analyzed the proteomic data and identified the differential proteins CD14 and CD11c, which are markers for monocytes, integrating both types of omics data allowed us to easily distinguish them. This explains why using multi-omics data can help us achieve more accurate cell clustering. By performing differential analysis on the cell clusters classified by scDRMAE, we found that the top differential features of each cluster have been validated as biological markers for certain cell types, further confirming the accuracy of the scDRMAE model in cell clustering. In addition, although some of the selected biomarkers have not been fully validated, by observing Fig. 8a and b, we can see significant differences between clusters. To more vividly display these differences, we visualized the selected differential genes in Fig. 8c and d, suggesting that these differential features may be potential biomarkers. Interestingly, we discovered that the differential protein CD127 selected from cluster 0 is translated from the differential gene IL7R (Zhang *et al.* 2022), indicating that the scDRMAE model has potential value and application prospects in exploring associations across different omics, promising to provide directions for biologists.

## 4 Discussion

Previous methods for cell clustering often relied on differences in gene expression data or signal values in epigenetic data to differentiate cell types. However, we believe that the dependencies between features within different omics often vary significantly among different cell types, yet this factor is rarely considered in cell clustering. Therefore, in this paper, we propose a deep learning method based on the scDRMAE model for integrating single-cell multi-omics data and clustering cells. scDRMAE approaches from the perspective of feature dependencies within different omics, using masked autoencoders to capture these dependencies across various omics and using a masking prediction mechanism to anticipate and compensate for dropout events. To dynamically integrate the low-dimensional representations of different omics data, we use an attention mechanism to learn the global structural information of the data, thereby dynamically allocating weights. To prevent network degradation, we use a structure similar to residual networks, enhancing the omics information from scRNA-seq, which is crucial for cell clustering, to prevent information loss during training.

Extensive experiments have demonstrated scDRMAE’s powerful performance in cell clustering, maintaining good results even under high dropout conditions. In addition, we conducted differential analysis on the cell clusters identified by scDRMAE, examining the top-ranked differential features to confirm their identities as biomarkers, further validating the effectiveness of scDRMAE in cell clustering. Moreover, for some unconfirmed differential features, by observing the UMAP plots, we noticed significant differences among various cell types, suggesting these features as potential biomarkers, providing guidance for biologists.

## Supplementary Material

btae599_Supplementary_Data

## Data Availability

You can access the open-source Python implementation of scDRMAE on GitHub at https://github.com/hongfeiZhang-source/scDRMAE.
