# Supplementary material for: scDRMAE: integrating masked autoencoder with residual attention networks to leverage omics feature dependencies for accurate cell clustering
Source: Bioinformatics. 2024 Oct 15;40(10):btae599. doi: 10.1093/bioinformatics/btae599 (PMC11513018; doi:10.1093/bioinformatics/btae599)
Supplement: btae599_Supplementary_Data [file btae599_supplementary_data.pdf]

Supplementary Table 1 ARI metrics of multi-omics datasets composed of scATAC-seq and scRNA-seq.

datasets	scDRMAE	scEMC	scMCs	Mocluster	Kmeans	scMAE	scRISE
human cell line mixture	**0.9635**	0.9165	0.888	0.4152	0.21	0.7504	0.6606
Mouse_brain	**0.4432**	0.2927	0.1115	0.2621	0.0883	0.3884	0.3098
MA-53	**0.4798**	0.0498	0.424	0.2703	0.2336	0.3916	0.2767
MA-54	**0.3225**	0.0844	0.299	0.164	0.2079	0.1683	0.15
MA-55	**0.3804**	0.0528	0.1878	0.2463	0.2001	0.3506	0.1751
MA-56	0.3418	0.0692	**0.3501**	0.2509	0.3061	0.3269	0.2244
BMMC-24	**0.7267**	0.6428	0.4444	0.5299	0.5555	0.6598	0.567
BMMC-49	**0.5808**	0.543	0.292	0.5029	0.3787	0.5124	0.4357
BMMC-41	**0.696**	0.6698	0.363	0.5448	0.476	0.6435	0.443
BMMC-36	**0.5629**	0.5405	0.2166	0.3789	0.3379	0.4773	0.4225

Note: The ARI values in the table represent the average from five-fold cross-validation

Supplementary Table 2 AMI metrics of multi-omics datasets composed of scATAC-seq and scRNA-seq.

datasets	scDRMAE	scEMC	scMCs	Mocluster	Kmeans	scMAE	scRISE
human cell line mixture	**0.9325**	0.8846	0.8834	0.4346	0.4685	0.7461	0.6755
Mouse_brain	**0.6118**	0.5073	0.215	0.45	0.2227	0.5698	0.4976
MA-53	**0.6751**	0.1504	0.6397	0.4716	0.4513	0.6247	0.5156
MA-54	**0.5752**	0.1942	0.4785	0.3554	0.3687	0.3164	0.3268
MA-55	**0.6057**	0.1682	0.3965	0.4269	0.3762	0.561	0.3416
MA-56	**0.5857**	0.1655	0.5524	0.4476	0.4229	0.5639	0.4123
BMMC-24	**0.8217**	0.7858	0.5819	0.7049	0.69	0.7906	0.727
BMMC-49	**0.7087**	0.681	0.4654	0.6556	0.5511	0.7009	0.6225
BMMC-41	**0.7942**	0.7881	0.527	0.6952	0.5988	0.7608	0.6573
BMMC-36	**0.7219**	0.6831	0.3248	0.5695	0.4718	0.6534	0.6061

Note: The AMI values in the table represent the average from five-fold cross-validation

Supplementary Table 3 NMI metrics of multi-omics datasets composed of scATAC-seq and scRNA-seq.

datasets	scDRMAE	scEMC	scMCs	Mocluster	Kmeans	scMAE	scRISE
human cell line mixture	**0.9474**	0.8865	0.892	0.4442	0.4791	0.7703	0.6811
Mouse_brain	**0.6485**	0.546	0.2725	0.4925	0.2668	0.6112	0.5438
MA-53	**0.698**	0.1715	0.6616	0.4988	0.4819	0.6509	0.5491
MA-54	**0.5923**	0.2115	0.4958	0.3749	0.3863	0.3225	0.3522
MA-55	**0.6226**	0.188	0.4157	0.4416	0.395	0.5796	0.369
MA-56	**0.6143**	0.1953	0.5794	0.475	0.4484	0.5933	0.4511
BMMC-24	**0.8296**	0.7977	0.5983	0.7132	0.6915	0.7999	0.739
BMMC-49	**0.7281**	0.7005	0.4909	0.6737	0.5748	0.7209	0.6477
BMMC-41	**0.8025**	0.7958	0.5424	0.7029	0.6182	0.7705	0.671
BMMC-36	**0.7627**	0.7237	0.3923	0.3789	0.5283	0.7041	0.6539

Note: The NMI values in the table represent the average from five-fold cross-validation

Supplementary Table 4 ARI metrics of multi-omics datasets composed of scADT-seq and scRNA-seq.

datasets	scDRMAE	scEMC	scMCs	Mocluster	Kmeans	scMAE	scRISE	Kmeans_ADT	scMAE_ADT
GSE148665	**0.9215**	0.8696	0.5035	0.2365	0.5932	0.571	0.3402	0.8274	0.685
BMNC	**0.7223**	0.6689	0.3663	0.3936	0.491	0.4789	0.3754	0.5196	0.56
Ncl	**0.4914**	0.2647	0.2243	0.2252	0.4098	0.4155	0.3272	0.1494	0.1045
Cambridge	**0.4124**	0.2231	0.1468	0.2185	0.2718	0.3408	0.2407	0.1116	0.1379
PBMC 10K	0.6694	0.14	0.5226	0.5069	0.4276	0.024	0.036	0.6119	**0.6995**

Note: The ARI values in the table represent the average from five-fold cross-validation

Supplementary Table 5 NMI metrics of multi-omics datasets composed of scADT-seq and scRNA-seq.

datasets	scDRMAE	scEMC	scMCs	Mocluster	Kmeans	scMAE	scRISE	Kmeans_ADT	scMAE_ADT
GSE148665	**0.9148**	0.9084	0.6644	0.4008	0.6859	0.6465	0.5054	0.9117	0.8428
BMNC	**0.7851**	0.6809	0.5798	0.5976	0.6935	0.704	0.5868	0.7483	0.7453
Ncl	**0.6996**	0.6053	0.4359	0.4732	0.6618	0.6586	0.5422	0.4003	0.2973
Cambridge	**0.6239**	0.5768	0.3603	0.4913	0.5634	0.614	0.4998	0.3508	0.4099
PBMC 10K	0.7855	0.1964	0.6468	0.6299	0.5564	0.0292	0.0328	0.701	**0.8091**

Note: The NMI values in the table represent the average from five-fold cross-validation

Supplementary Table 6 AMI metrics of multi-omics datasets composed of scADT-seq and scRNA-seq.

datasets	scDRMAE	scEMC	scMCs	Mocluster	Kmeans	scMAE	scRISE	Kmeans_ADT	scMAE_ADT
GSE148665	**0.9114**	0.9061	0.652	0.3773	0.6741	0.6319	0.4871	0.9083	0.837
BMNC	**0.7819**	0.6785	0.5737	0.5903	0.687	0.6978	0.5785	0.743	0.7398
Ncl	**0.6942**	0.6008	0.4259	0.4579	0.6582	0.6519	0.5341	0.3884	0.2846
Cambridge	**0.6108**	0.5656	0.3392	0.4745	0.5544	0.6001	0.4824	0.3263	0.3877
PBMC 10K	0.7837	0.19	0.6438	0.6268	0.5522	0.0208	0.0239	0.6984	**0.8075**

Note: The AMI values in the table represent the average from five-fold cross-validation

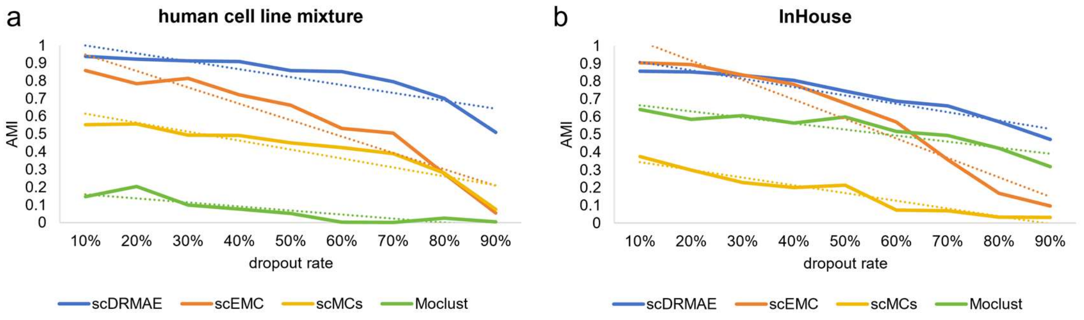

Supplementary Figure 1 AMI various methods at different dropout rates across two datasets. (a) AMI different methods on the human cell line mixture dataset, (b) AMI different methods on the InHouse dataset

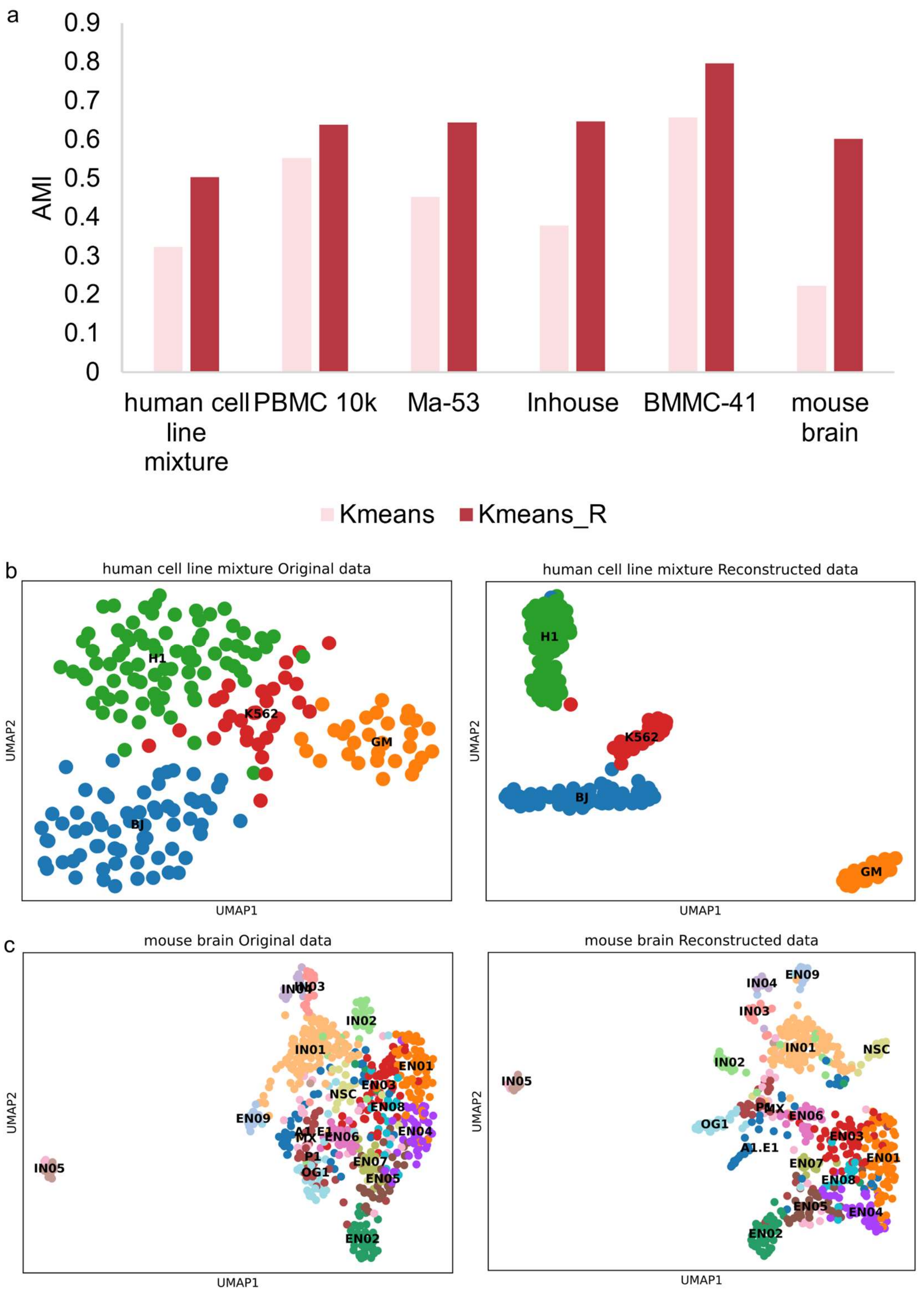

Supplementary Figure 2 Comparison of mouse brain and human cell line mixture datasets before and after imputation. (a)AMI before and after imputation on different datasets. (b) Shows the UMAP plot of the human cell line mixture dataset before and after scRNA-seq imputation, where the left plot represents the original data and the right plot represents the imputed data. (c) Displays the UMAP plot of the mouse brain dataset before and after scRNA-seq imputation using scDRMAE, with the left plot showing the original data and the right plot showing the imputed data. It can be observed that after imputation with scDRMAE, the distribution differences between different cell types are more

pronounced, with similar cell types clustering together and different cell types separating.

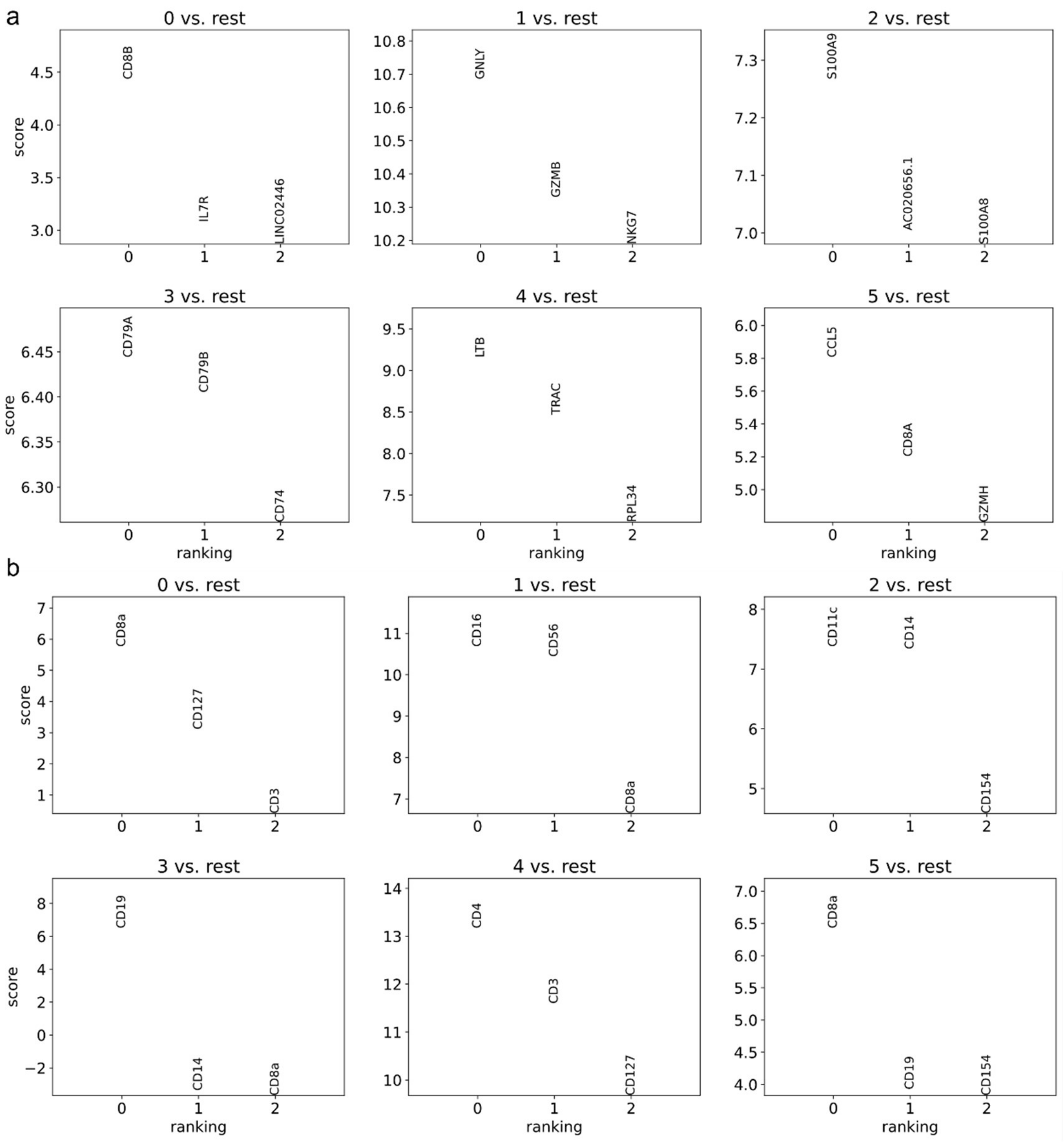

Supplementary Figure 3 Differential features of distinct clusters identified by scDRMAE in the InHouse dataset. (a) The top 3 differential genes of the different clusters identified by scDRMAE. (b) The top 3 differential proteins of the different clusters identified by scDRMAE.

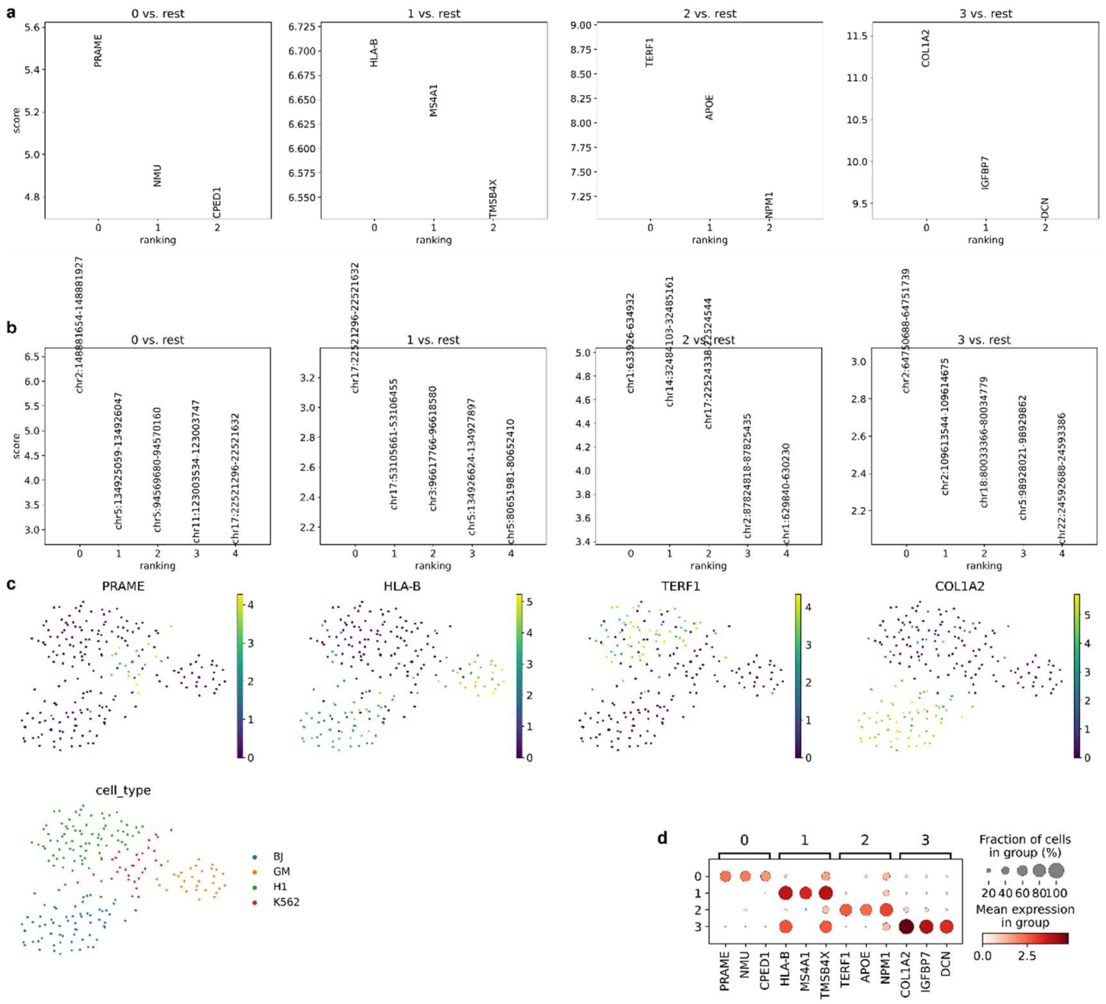

Supplementary Figure 4 Differential genes and their visualization for distinct clusters in the human cell line mixture dataset. (a) The top 3 differential genes for the different clusters identified by scDRMAE. (b) The top 5 differential chromatin accessible regions for the different clusters identified by scDRMAE. (c) Visualization of differential genes for the different clusters on the UMAP plot. (d) A bubble chart displaying the differential genes for the different clusters.

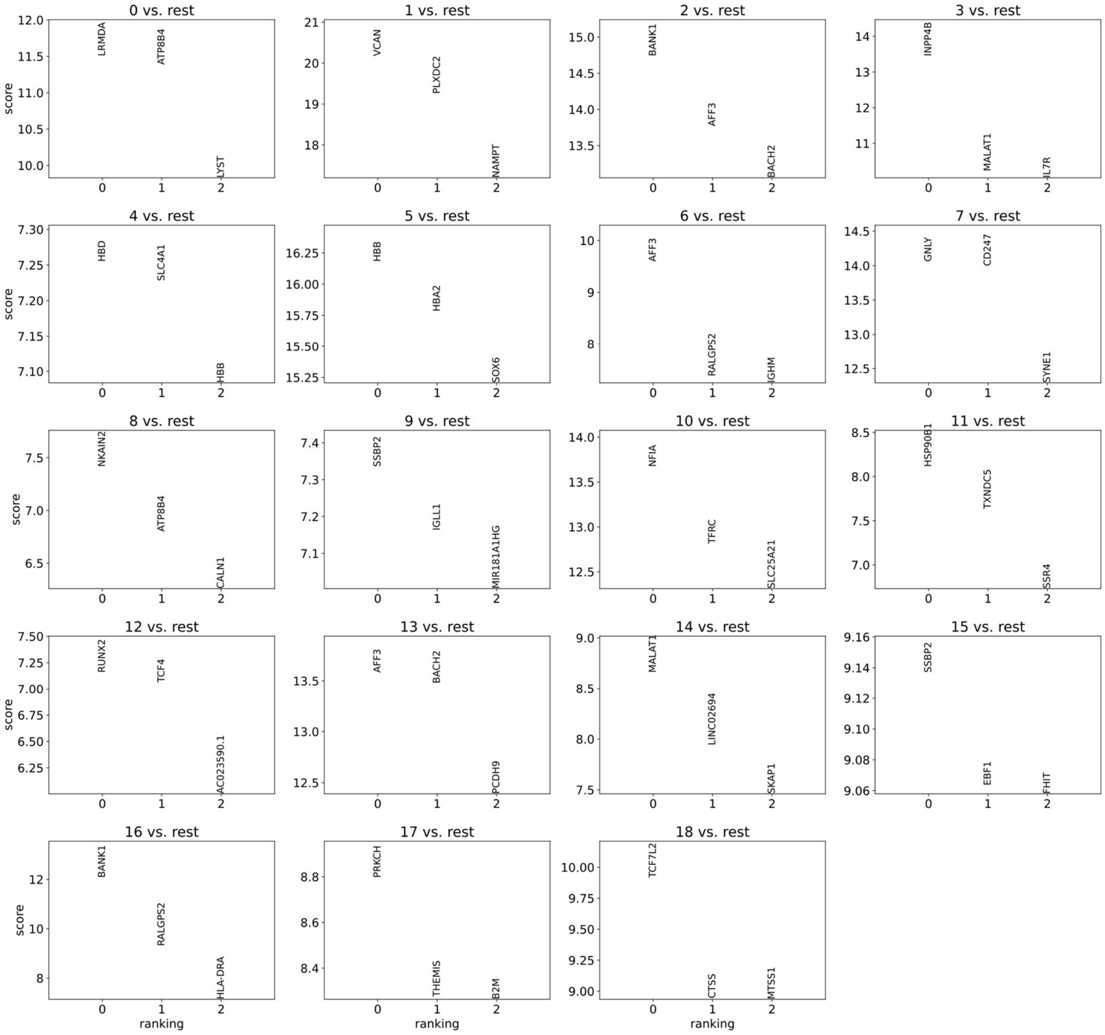

Supplementary Figure 5 The top 3 differential genes of different clusters in the BMMC-41 dataset.

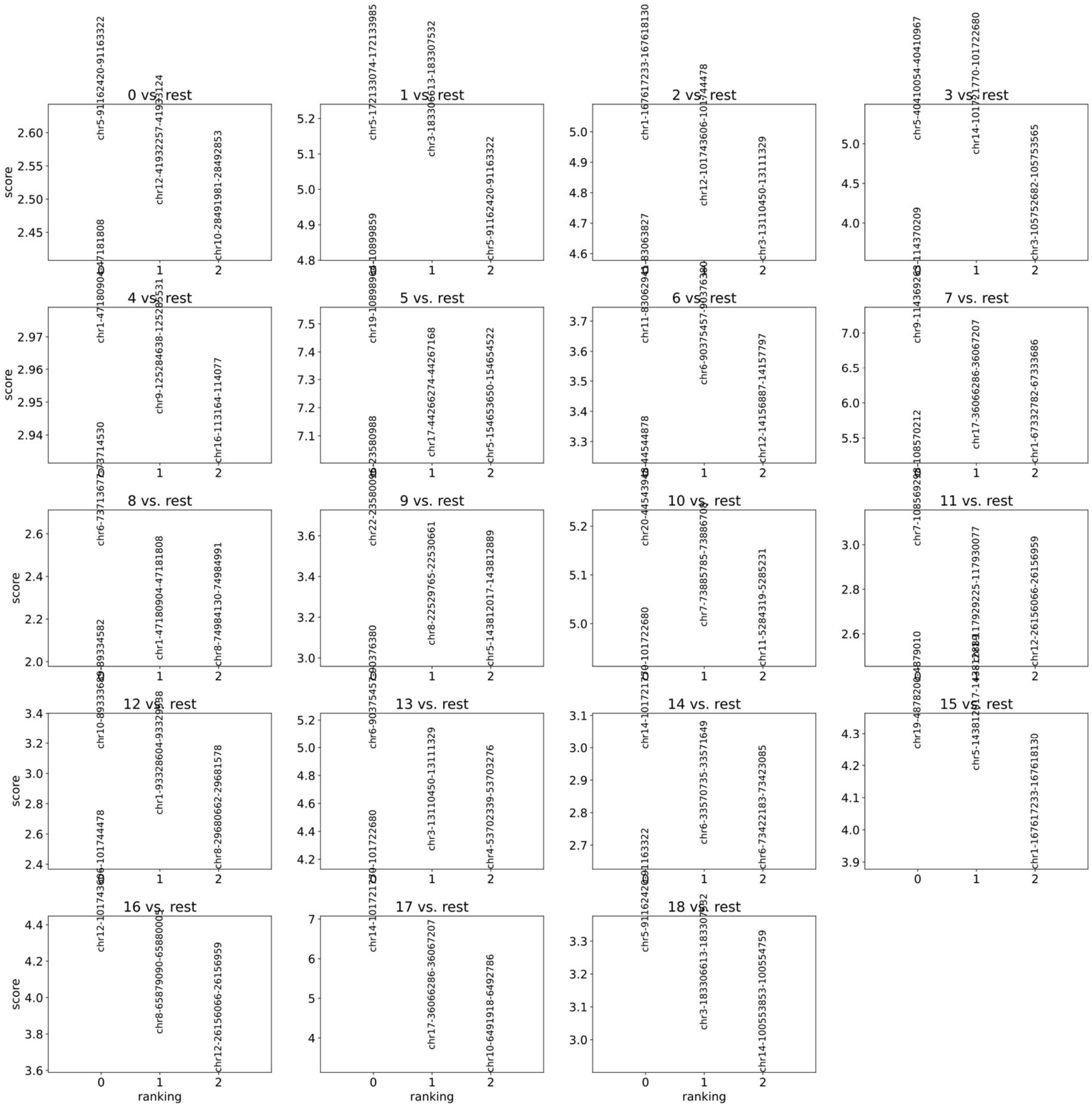

Supplementary Figure 6 The top 5 differential chromatin accessible regions of different clusters in the BMMC-41 dataset.

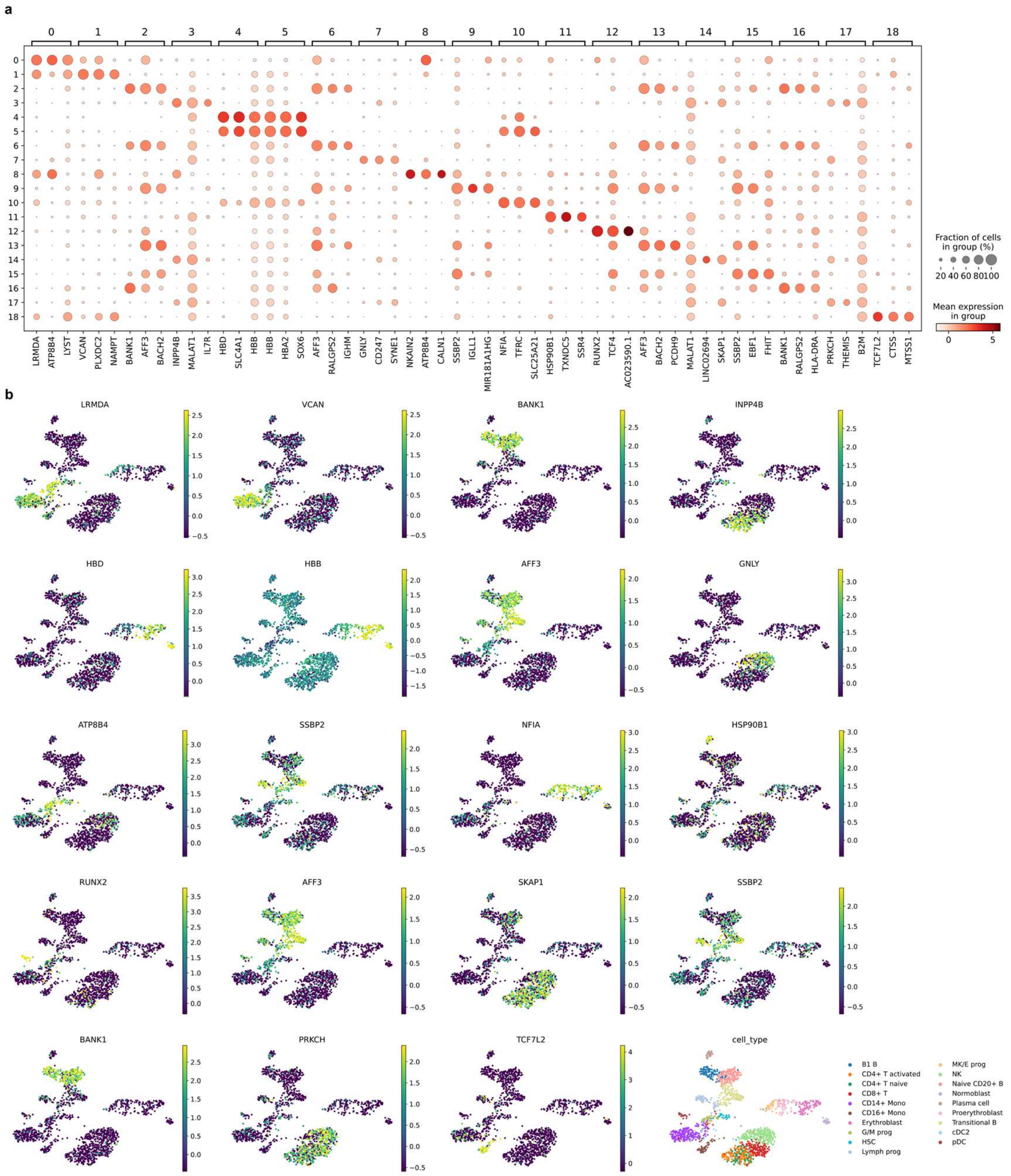

Supplementary Figure 7 Visualization of differential genes for different clusters in the BMMC-41 dataset. (a) A bubble chart displaying the differential genes for different clusters. (b) Visualization of differential genes for different clusters on the UMAP plot.

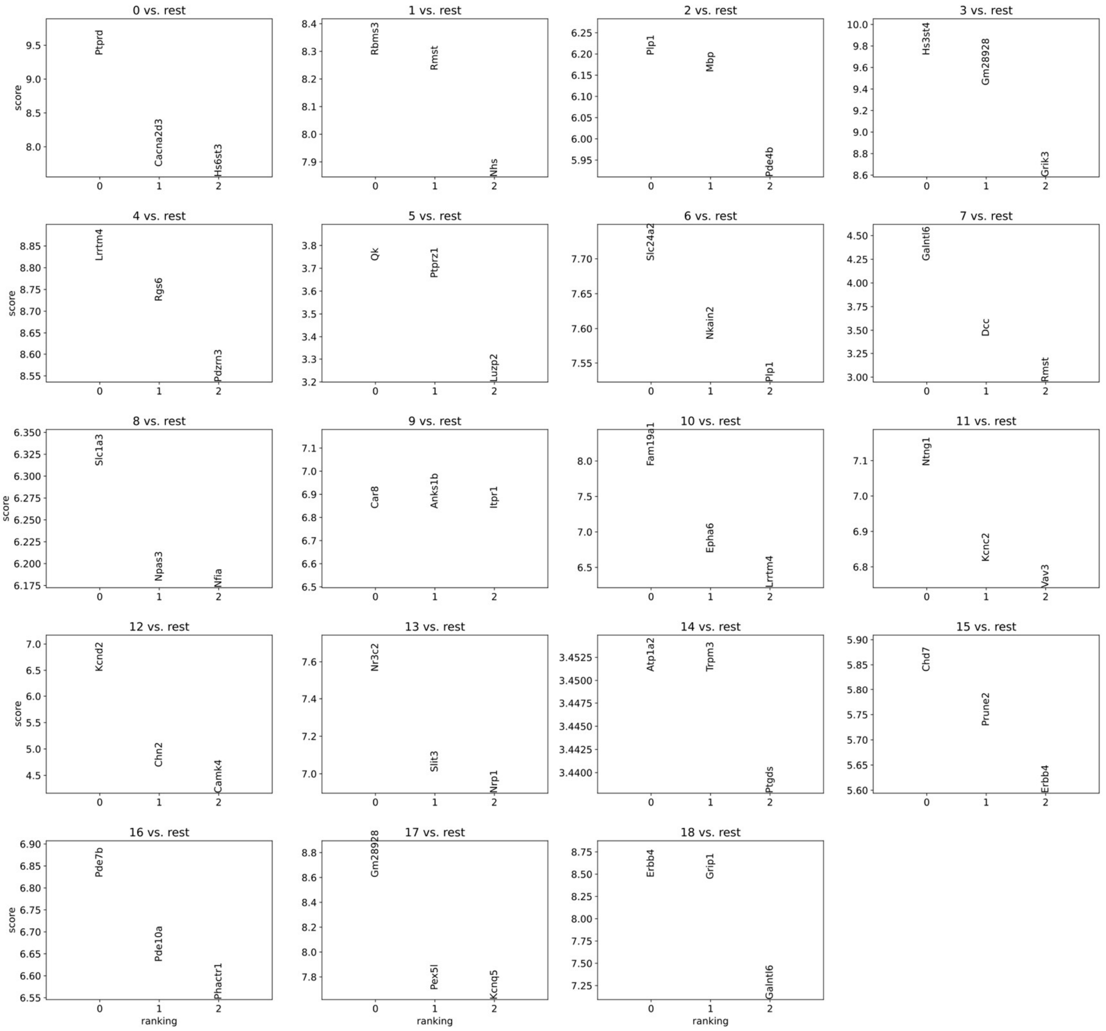

Supplementary Figure 8 The top 3 differential genes of different clusters in the mouse brain dataset.

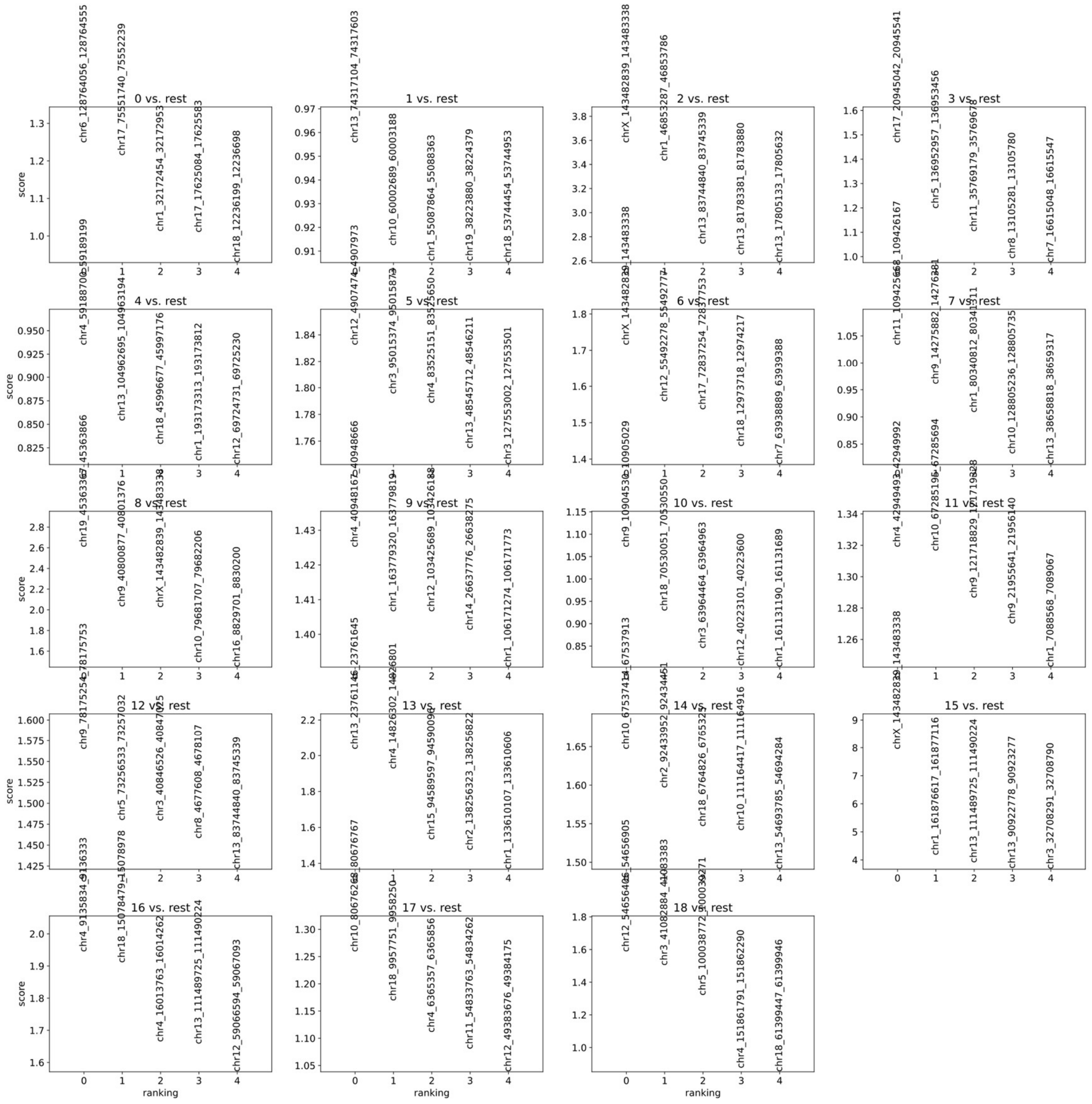

Supplementary Figure 9 The top 5 differential chromatin accessible regions of different clusters in the mouse brain dataset.

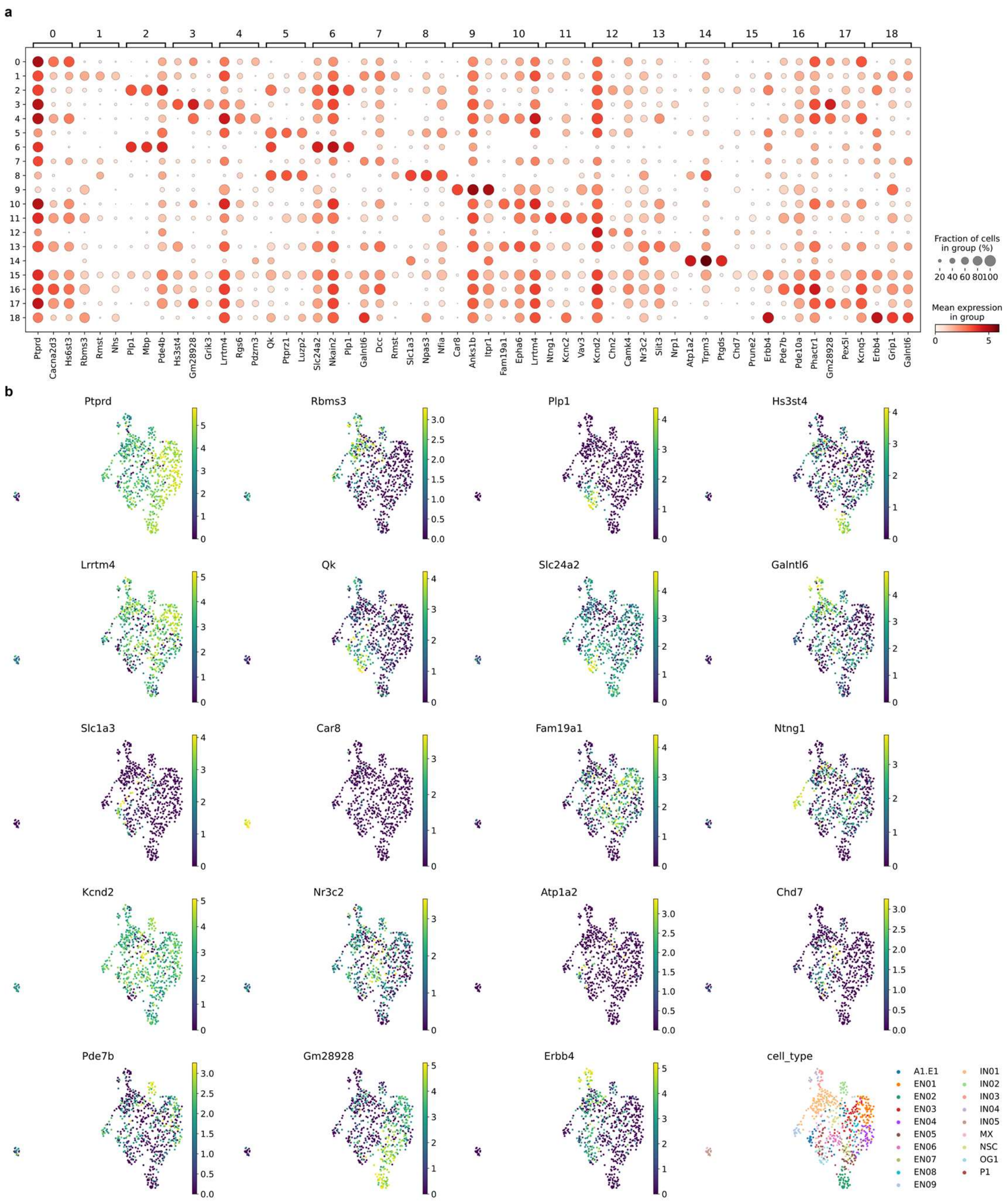

Supplementary Figure 10 Visualization of differential genes for different clusters in the mouse brain dataset. (a) A bubble chart displaying the differential genes for different clusters. (b) Visualization of differential genes for different clusters on the UMAP plot.
